# A high-throughput screen to identify novel small molecule inhibitors of the Werner Syndrome Helicase-Nuclease (WRN)

**DOI:** 10.1371/journal.pone.0210525

**Published:** 2019-01-09

**Authors:** Joshua A. Sommers, Tomasz Kulikowicz, Deborah L. Croteau, Thomas Dexheimer, Dorjbal Dorjsuren, Ajit Jadhav, David J. Maloney, Anton Simeonov, Vilhelm A. Bohr, Robert M. Brosh

**Affiliations:** 1 Laboratory of Molecular Gerontology, National Institute on Aging, National Institutes of Health, Baltimore, Maryland, United States of America; 2 National Center for Advancing Translational Sciences, National Institutes of Health, Rockville, Maryland, United States of America; University of Iowa, UNITED STATES

## Abstract

Werner syndrome (WS), an autosomal recessive genetic disorder, displays accelerated clinical symptoms of aging leading to a mean lifespan less than 50 years. The WS helicase-nuclease (WRN) is involved in many important pathways including DNA replication, recombination and repair. Replicating cells are dependent on helicase activity, leading to the pursuit of human helicases as potential therapeutic targets for cancer treatment. Small molecule inhibitors of DNA helicases can be used to induce synthetic lethality, which attempts to target helicase-dependent compensatory DNA repair pathways in tumor cells that are already genetically deficient in a specific pathway of DNA repair. Alternatively, helicase inhibitors may be useful as tools to study the specialized roles of helicases in replication and DNA repair. In this study, approximately 350,000 small molecules were screened based on their ability to inhibit duplex DNA unwinding by a catalytically active WRN helicase domain fragment in a high-throughput fluorometric assay to discover new non-covalent small molecule inhibitors of the WRN helicase. Select compounds were screened to exclude ones that inhibited DNA unwinding by other helicases in the screen, bound non-specifically to DNA, acted as irreversible inhibitors, or possessed unfavorable chemical properties. Several compounds were tested for their ability to impair proliferation of cultured tumor cells. We observed that two of the newly identified WRN helicase inhibitors inhibited proliferation of cancer cells in a lineage-dependent manner. These studies represent the first high-throughput screen for WRN helicase inhibitors and the results have implications for anti-cancer strategies targeting WRN in different cancer cells and genetic backgrounds.

## Introduction

There has been a significant interest in characterizing the molecular and cellular functions of the *WRN* gene product that is defective in the hereditary accelerated aging disorder WS [1]. *WRN* encodes a protein with dual catalytic activities, a 3ʹ to 5ʹ DNA helicase and a 3ʹ to 5ʹ DNA exonuclease [2, 3]. An extensive number of studies have examined WRN DNA substrate specificity as well as its protein interactions [4–9]. Collectively, these studies build upon the findings from cell-based assays which suggest that WRN is a multi-tasking enzyme with pleiotropic roles in cellular nucleic acid metabolic pathways including replication, DNA repair, recombination, and transcription. Although these investigations from multiple laboratories have proven to be very informative for gaining insight to WRN’s roles in maintenance of genomic stability mediated by its functions at telomeres, stalled forks, and key DNA recombination intermediates, the enzyme is still enigmatic in terms of establishing direct relationships between its molecular functions and cellular pathways critical for genome homeostasis and suppression of gerontological phenotypes characteristic of the accelerated aging disorder WS.

A relatively new approach for studying the functions and pathways of DNA helicases, like WRN, is to identify and characterize small molecule inhibitors of their DNA unwinding activities to interrogate their cellular pathways [10, 11]. The first human DNA helicase inhibitor discovered was one that specifically inhibited WRN-catalyzed DNA unwinding and acted in a WRN-dependent manner in cell-based assays [12]. This compound, designated NSC 19630, was initially identified from a National Cancer Institute (NCI) library of compounds with diverse chemotypes by *in vitro* radiometric strand displacement WRN helicase assays. NSC 19630, and a structurally related compound NSC 617145 [13], were determined to be biologically active in human cell culture experiments and have been used to interrogate the role of WRN in the cellular response to DNA damage or replication stress. It is now generally believed that the WRN helicase inhibitors, as well as other helicase and DNA repair inhibitors, will prove to be highly valuable for providing insight into cellular genome stability maintenance pathways as well as developing potential therapeutic synthetic-lethal strategies, particularly those based on DNA damaging chemotherapy drugs or radiation [14].

In the current study, we set out to establish a high-throughput WRN helicase activity assay that avoids the use of radioactivity to greatly expand the potential number of compounds that could be tested in the initial biochemical screen. The development of such a large-scale *in vitro* strategy may be advantageous compared to the more traditional and smaller scale screens that have been performed up to this point to identify WRN helicase inhibitors [15]. We describe the experimental methodologies for development of the screen and the characterization of several novel compounds that displayed an inhibitory effect on WRN-catalyzed DNA unwinding. Two of these newly discovered compounds that inhibit WRN helicase activity inhibit cell proliferation in a manner that is dependent on p53 and/or telomerase status. These results provide a new avenue of exploration for the identification and characterization of WRN helicase inhibitors that will be useful to the community engaged in efforts to target pathways of nucleic acid metabolism for basic science and possibly therapeutic approaches.

## Materials and methods

### Cell lines

U2-OS cells were obtained from ATCC (HTB96). HeLa 1.2.11 cells were obtained from the laboratory of Titia de Lange (The Rockefeller University). Both cell lines were grown in DMEM with 10% Fetal Bovine Serum (FBS) and Penicillin/Streptomycin (Pen/Strep) antibiotics.

### Recombinant proteins

GST-WRN_500-946_ for the low volume screening assay (96 well plate) was purified from E. coli as described [16]. GST-WRN_500-946_ for the high throughput screen was purified (S1 Fig) from insect cells and dialyzed into 100 mM Tris (pH 8.0), 150 mM NaCl, 10% glycerol as described [17]. Full length recombinant WRN [18], BLM [19], and FANCJ [20] proteins were purified from insect cells as described and representative protein gels are shown (S1 Fig).

### DNA substrate preparation

For all of the screening reactions, a fluorescent forked DNA substrate FORKF was prepared by annealing equal amounts of OLIGOA-BHQ2 (TTTTTTTTTTTTTTTTTTTTTTTTTTTTTTCGTACCCGATGTGTTCGTTC-BHQ2) and OLIGOB-TAMRA (TAMRA-GAACGAACACATCGGGTACGTTTTTTTTTTTTTTTTTTTTTTTTTTTTTT) in equal amounts, boiling for 5 min and allowing the oligos to slowly cool to room temperature in the presence of 50 mM NaCl. For radioactive confirmation assays, ^32^P-labeled FORKR was prepared by labeling 10 pmol of DC26 (5ʹ-TTTTTTTTTTTTTTTTTTTTTTCCCAGTAAAACGACGGGCAGTGC-3ʹ) with 30 μCi γ^32^P-adenosine triphosphate (ATP) and T4 polynucleotide kinase (T4-PNK), passing through a G-25 spin column (GE Healthcare Life Sciences) and annealing to 25 pmol of TSTEM25 (5ʹ-GCACTGGCCGTCGTTTTACGGTCGTGACTGGGAAAACCCTGGCG-3ʹ) by boiling for 5 min followed by slow cooling to room temperature in the presence of 50 mM NaCl.

### 96 well screening assay

Forty-three μL of reaction buffer (25 mM Tris-HCl (pH 8.0), 5 mM NaCl, 2 mM MgCl_2_, 1 mM dithiothreitol (DTT), 0.01% Tween- 20, and 2.5 μg/ml calf thymus DNA, including enzyme (24–96 nM GST-WRN_500-946_) and small molecule diluent (dimethyl sulfoxide (DMSO), 1 μL volume), were dispensed into a 96-well Greiner black assay plate. The plates were incubated for 15 min at room temperature, and then 1 μL of FORKF (200 nM DNA final concentration) and 5 μL ATP (2 mM final concentration) were added to start the reaction. The plate was transferred into a Fluorstar Optima plate reader where the reaction progress was measured in fluorescence mode (reads at 0, 5, 15, 30, 45 and 60 min) at 25°C using fluorescence optics (excitation filter 544 nm, emission filter 590 nm). Enzyme-free controls, no-ATP control, and single-stranded DNA (unquenched) control were included for validation of assay. Fluorescence Units were corrected for each dataset based on initial fluorescence reading at 0 min.

### High-throughput screening assay

The Library of Pharmacologically Active Compounds (LOPAC^1280^) is available from Sigma-Aldrich and contains 1280 approved drugs, highly selective small molecule modulators of individual proteins and pathways, as well as naturally occurring compounds with widely described biological effects. The collection of 350,000 small molecules was assembled through aggregation of diverse chemical scaffolds from multiple vendor sources, with compounds accepted based on compliance with the Lipinsky Rule of 5, purity and identity of the delivered material, and adequate solubility in DMSO, under the auspices of the Molecular Libraries Initiative of the NIH Common Fund (https://commonfund.nih.gov/molecularlibraries/index). The structures of the members of this collection are available in the PubChem portal https://pubchem.ncbi.nlm.nih.gov/. For both the 350,000 small molecule collection and the LOPAC^1280^ screens, 3 μL of GST-WRN_500-946_ or reaction buffer only were dispensed using a solenoid-valve nanoliter dispenser into a black 1536-well plate (Greiner). Twenty three nL of each serially diluted compound (0.08–50 μM for the LOPAC screen and 0.003–114 μM for the larger library screen) or control were added to each well using a Kalypsys pintool equipped with a 1536-pin array. Plates were incubated at room temperature for 15 min, followed by initiation of the reactions via the addition of 1 μL of substrate (100 nM FORKF and 2 mM ATP final concentrations). Plates were transferred to a ViewLux high-throughput CCD imager and fluorescence was measured in kinetic mode using an excitation filter of 525 nm and emission filter of 598 nm.

### Quantitation of screening data

Zʹ factor, a measure for assay quality control was calculated using the formula *Z*′ = 1−(3*σS*^+^+3*σS*^−^)∕|*μS*^+^−*μS*^−^| as described previously [21]. In this formula, 3*σS*^+^ represents 3 standard deviations from the mean of positive signals while 3*σS*^−^ represents 3 standard deviations from the mean of negative signals. *μS*^+^ and *μS*^−^ represent the mean of positive signals and negative signals respectively. Percent activity was derived using in-house software (http://tripod.nih.gov/curvefit/). Dose-response curves were classified as described previously [22].

### Radiometric helicase inhibitor assay

Fifteen μL of reaction buffer was added to a microcentrifuge tube followed by the addition of 1 μL of selected compounds (0.5–100 μM final concentration) or DMSO. One μL of WRN (full length), RECQ1, FANCJ, BLM or control was added to each reaction and incubated at room temperature for 15 min. Three μL of substrate (0.5 nM FORKR and 2 mM ATP final concentrations) were added to all reactions which were incubated at 37°C for 15 min. Reactions were stopped with the addition of 20 μL of 2X STOP dye containing EDTA, glycerol, bromphenol blue and xylene cyanol. Samples were electrophoresed for 1.5 hr at 200 V on a 12% non-denaturing PAGE gel in 1X TBE Buffer.

### Radiometric helicase inhibitor reversibility assay

WRN (full length, 100 nM concentration) was incubated with each small molecule inhibitor at a concentration 10-fold higher than the IC_50_ for that inhibitor, in a 10 μL reaction at room temperature. 1.5 μL of this reaction was added to 148.5 μL of reaction salts with 2 mM ATP, 0.5 nM FORKR reducing the full length WRN concentration to 1 nM and the drug concentration to 10-fold less than its IC_50_. Reactions were placed at 37°C and 20 μL of reaction were removed at specific intervals (0–16 min) and quenched with 20 μL of 2X STOP dye containing EDTA, glycerol, bromphenol blue and xylene cyanol. Samples were electrophoresed for 1.5 hr at 200 V on a 12% non-denaturing PAGE gel in 1X TBE Buffer.

### Cell proliferation assay

WRN inhibitors were diluted in DMSO to a concentration 100-fold higher than their final concentrations. U2-OS cells in culture were counted, spun down and resuspended to 35,000 cells/mL in DMEM with 10% FBS and Pen/Strep antibiotic. For each cell condition, 15 μL of DMSO or inhibitor was added to 1.5 ml of U2-OS cells in DMEM or DMEM alone. One hundred μL of each cell condition was plated in triplicate on each of four 96-well plates representing days 0–3. Ten μL of WST-1 cell proliferation reagent (Roche) was added to the day 0 plate and readings were taken 2 hr later using a plate reader set to 450 nm. WST-1 cell proliferation reagent was added to plates on days 1–3 and readings were taken after 2 hr incubation. Percent cell proliferation was normalized to cells in the presence of DMSO with the background OD_450_ of DMEM with each drug subtracted out.

## Results

### Development of a low-volume fluorometric helicase assay

**Fig 1:**
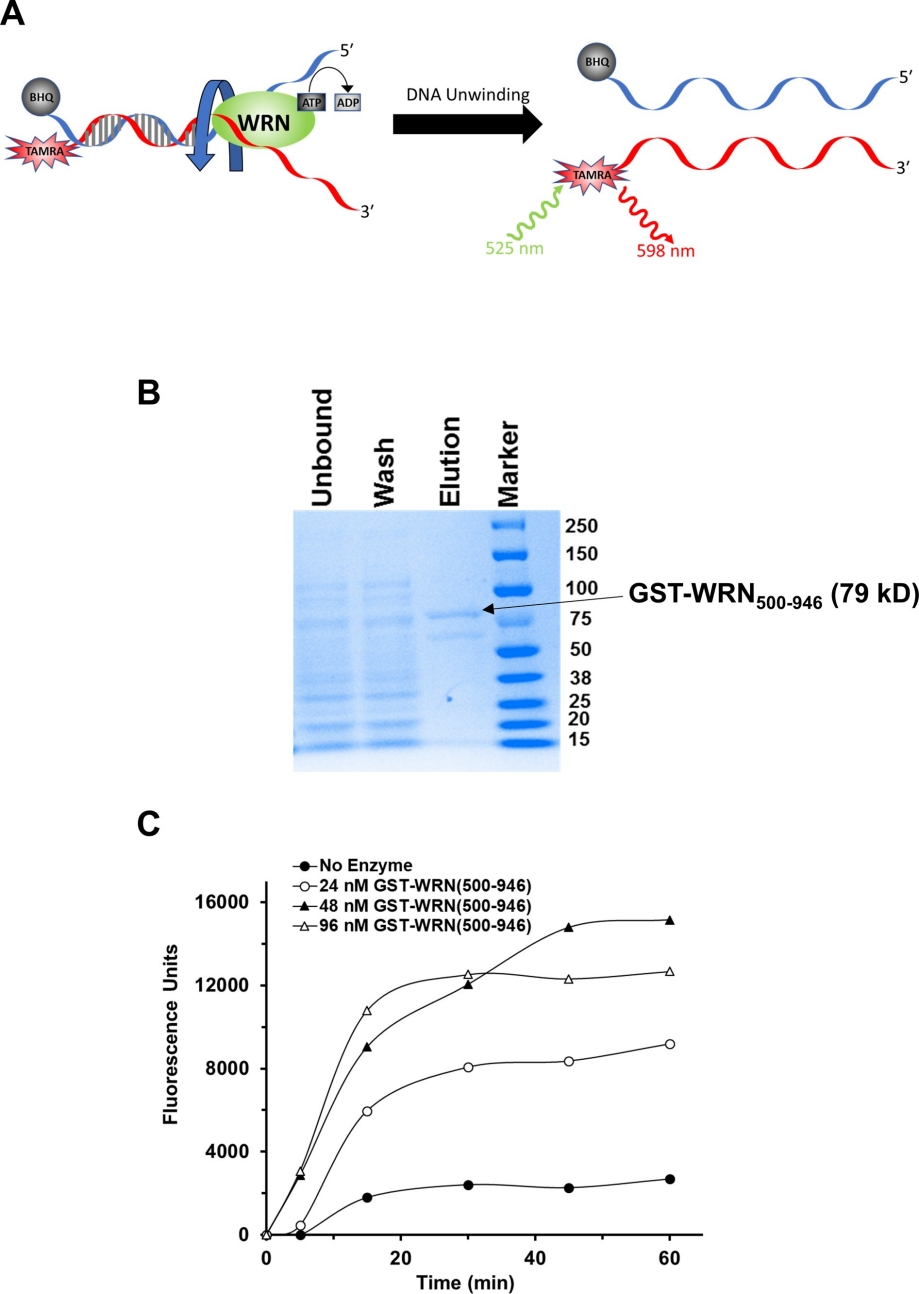
Fluorometric helicase assay development. **(A)** Depiction of fluorescently labeled forked DNA substrate for high-throughput helicase assays (FORKF). **(B)** Coomassie-stained gel showing purified GST-WRN_500-946_. The arrow indicates the band representing the GST-WRN_500-946_ fragment and its size in kD. **(C)** Graph of unwinding of the fluorescent forked DNA substrate FORKF (200 nM) by GST-WRN_500-946_ (0–96 nM) 0–60 min after initiating the reaction with ATP (2 nM final concentration) and FORKF DNA substrate in 96-well black plates. Background fluorescence recorded at T = 0 min was subtracted out from all time points for each protein concentration.

To develop a high-throughput fluorescent assay for assessing helicase activity, we needed to start by determining the best fluorophore, reaction conditions, and concentrations of reagents in a low-volume assay. Based on a similar study with BLM helicase [23], and a DNA substrate we have used to measure DNA unwinding by WRN and other helicases [6], we employed a 20 bp forked DNA duplex with 30 nucleotide tails with TAMRA and Black Hole Quencher 2 (BHQ2) moieties at the end of the duplex region (Fig 1A) for measuring WRN helicase activity. We also needed a method for production of catalytically active recombinant WRN helicase protein that could be scaled up to make 100 milligrams of protein for the high-throughput screen. For this, we purified a functional GST-tagged WRN helicase domain (amino acids 500–946, Fig 1B) which is small enough to be able to be produced at higher concentrations when compared to full length WRN protein, using a bacterial expression plasmid from previous work [16].

**Fig 2:**
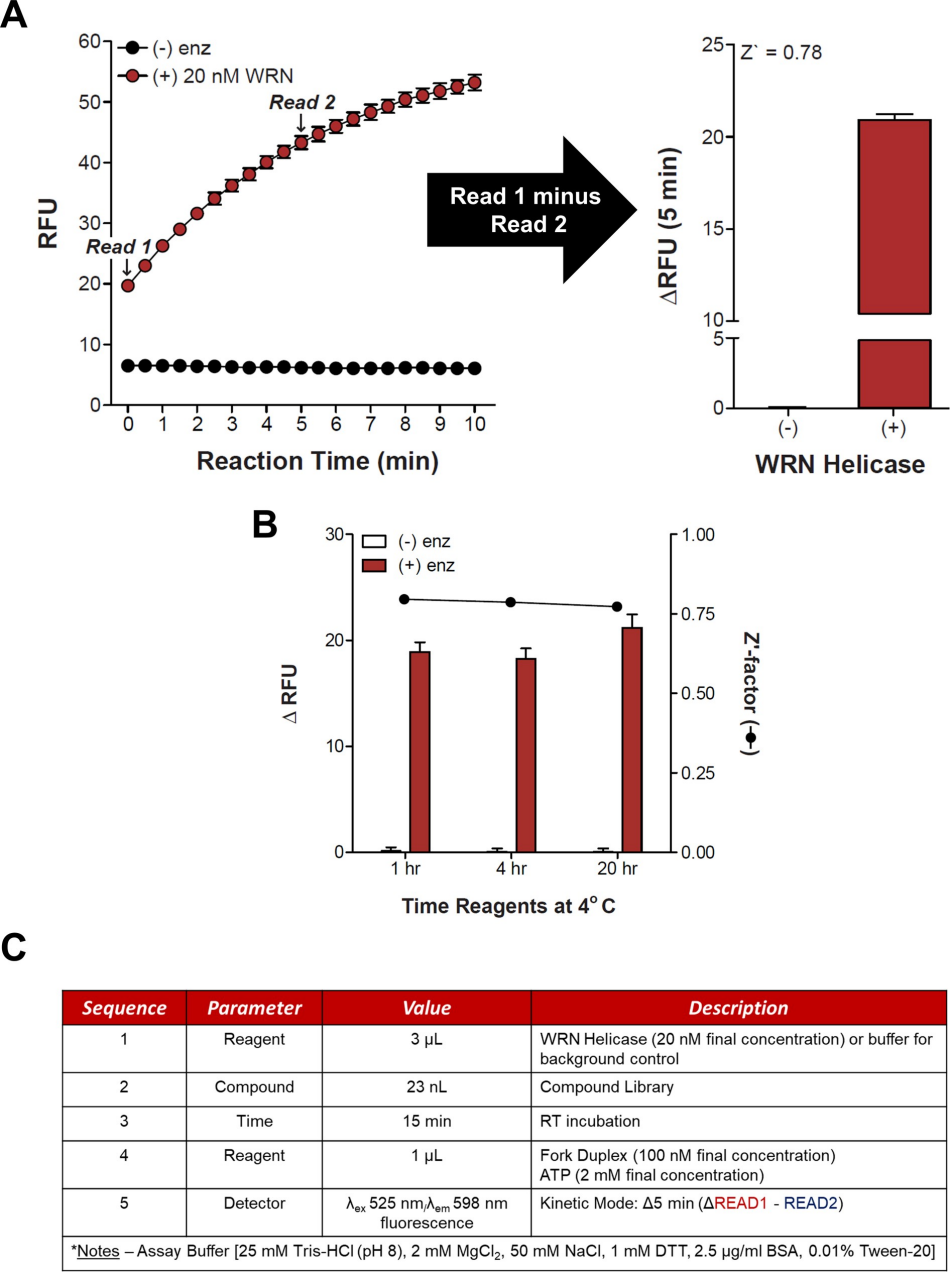
Fluorometric high-throughput helicase assay testing. **(A)** Graph of 20 nM E. coli-purified GST-WRN_500-946_ unwinding of FORKF DNA substrate (100 nM) compared to the absence of enzyme from 0–10 min after reaction initiation, and a bar graph showing change in fluorescence between time points T = 0 and T = 5 min and the corresponding Z’-factor which was calculated at described in Materials and Methods. (-) enz and (+) enz refer to the absence or presence of the GST-WRN fragment in the reaction. Reactions were performed in 1536-well black plates. **(B)** GST-WRN_500-946_ helicase activity (bars, change in fluorescent units) measured on the FORKF DNA substrate (100 nM) in the presence of 2 mM ATP after reagents were stored at 4° C for 1, 4 or 20 hr. The Z’-factor was calculated for each storage timepoint (line with closed circles). (-) enz and (+) enz refer to the absence or presence of the GST-WRN fragment in the reaction. Reactions were performed in 1536-well black plates. **(C)** High-throughput GST-WRN_500-946_ helicase assay reaction conditions and steps as determined from assay development.

Fluorescent readings were taken at several time points following the addition of FORKF (200 nM final concentration) and ATP (2 mM final concentration) to GST-WRN_500-946_ (0–96 nM), which had been preincubated at room temperature for 15 min to simulate binding to a small molecule. Background-subtracted fluorescence was plotted against time for each concentration of GST-WRN_500-946_ tested (Fig 1C). We could detect an increase in fluorescence for each concentration of GST-WRN_500-946_, at all the time points when compared to DNA substrate in the absence of enzyme. This proof of concept allowed us to do an initial test with instrumentation more closely resembling what would be used for the high throughput screen. In these assays, a kinetics assay was conducted to determine the minimum amount of time to run the reactions where a statistically significant amount of fluorescence measured due to GST-WRN_500-946_ (20 nM) unwinding of the fluorescent FORKF DNA substrate (100 nM) could be detected above background signal obtained from a reaction lacking enzyme (Fig 2A). It was determined that a reaction time of 5 min was sufficient with a Zʹ-factor of 0.78 (Fig 2A). The WRN helicase fragment was also tested for stability at 4° C for up to 20 hr by measuring and comparing its activity 1, 4 and 20 hr after placing at 4° C (Fig 2B): the demonstrated stability supported the planned large-scale automated screening campaign. Based on the preliminary work, reactions conditions were set for running the high throughput assay (Fig 2C). To ensure we could detect inhibition of GST-WRN_500-946_ helicase activity in the screen we set up a pilot run of the relatively small LOPAC^1280^ library.

### Screening of the LOPAC^1280^ library for WRN inhibitors for assay optimization and scalability

**Fig 3:**
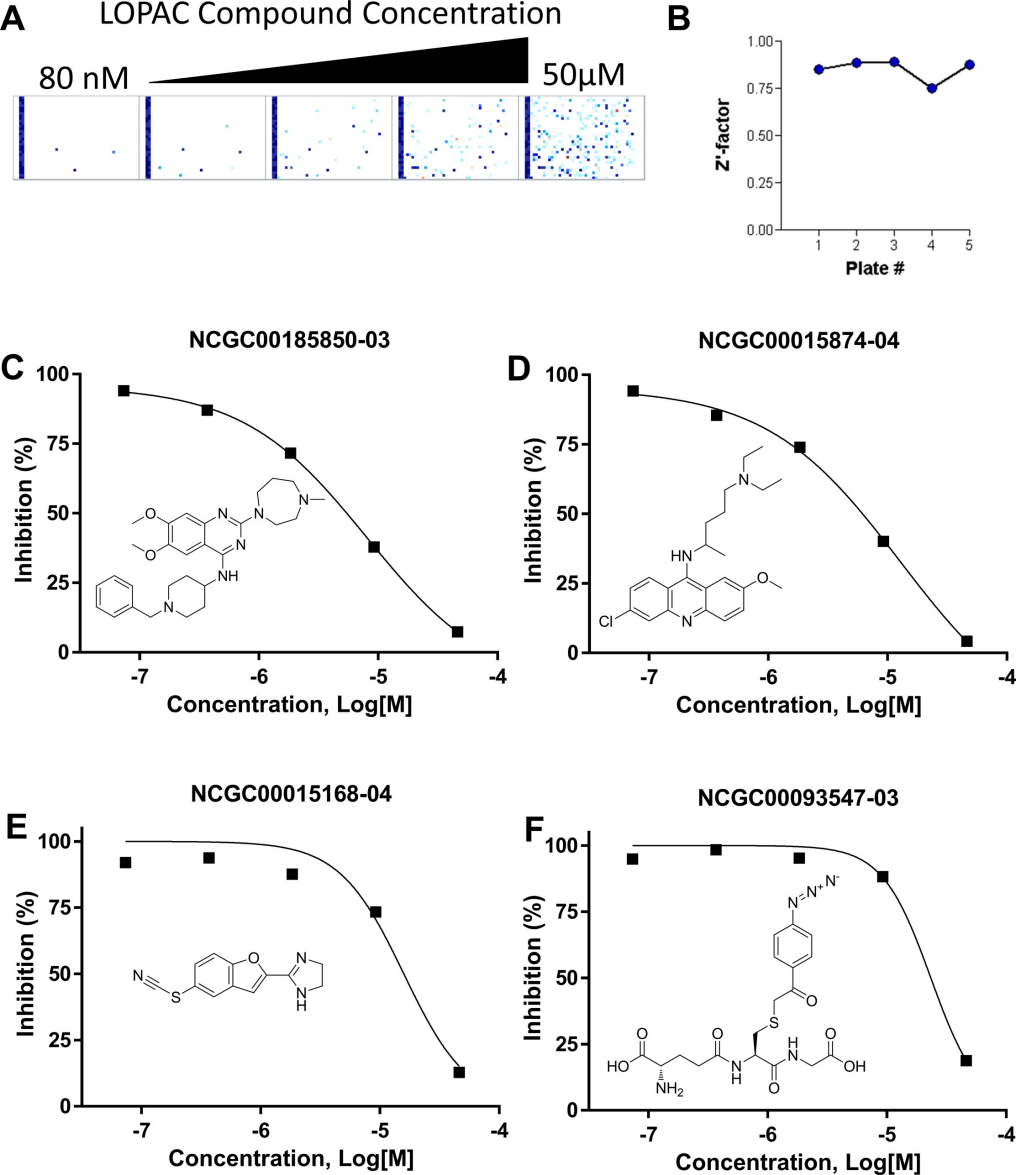
Screening of the LOPAC^1280^ library for WRN inhibitors. **(A)** Activity heatmap representation of the 1536-well plates containing the LOPAC^1280^ screen compounds, each plate rectangle corresponding to a different concentration (80 nM, 400 nM, 2 μM, 10 μM and 50 μM) of compound. Individual dots represent inhibition of GST-WRN_500-946_ (20 nM) unwinding of the FORKF DNA substrate (100 nM) by a compound at that particular concentration. **(B**) Z’-factor for each plate (concentration) in the LOPAC^1280^ screen. **(C-F)** Graphs showing inhibition of GST-WRN_500-946_ (20 nM) helicase activity on the FORKF DNA substrate (100 nM) by different molecules and the structures of those molecules. Concentration is in log units.

The next phase of the study was to utilize the LOPAC^1280^ library. The library consists of 1280 pharmacologically relevant drugs and other structures with known biological activities, providing a number of potential compounds for optimizing and validating the high-throughput assay. Five different 1536-well plates were set up, each plate containing a different concentration (80 nM to 50 μM) of all 1280 compounds as described in the Materials and Methods section, representing a dose-response curve also referred to as quantitative high-throughput screening (qHTS) [22]. Compounds that inhibited GST-WRN_500-946_ helicase (20 nM) activity on the FORKF DNA substrate (100 nM) are indicated by dots in each plate (rectangles, Fig 3A). The results obtained from each plate yielded a Zʹ-factor between 0.7 and 1 (Fig 3B) indicating that the assay strength was good throughout this pilot screen. Several compounds from the LOPAC screening library were active in inhibiting WRN helicase activity. Four compounds showing two distinct inhibition curves are shown as examples (Fig 3C–3F).

**Fig 4:**
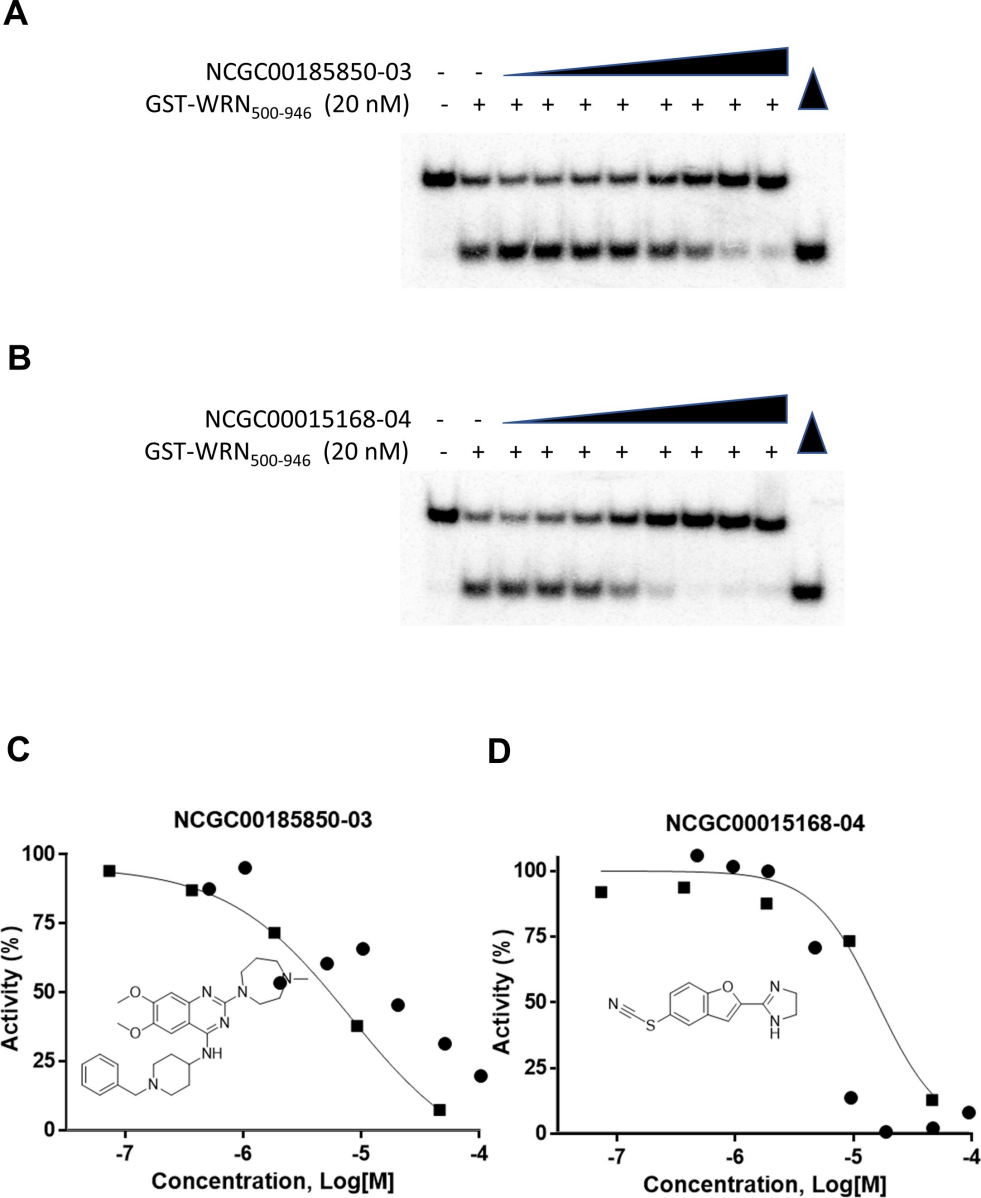
Comparison of Radiometric and high-throughput fluorescent helicase assays. **(A)** Gel image showing inhibition of GST-WRN_500-946_ (20 nM) unwinding of the radiolabeled FORKR DNA substrate (0.5 nM) by increasing concentrations of NCGC00185850-03. **(B)** Gel image showing inhibition of GST-WRN_500-946_ (20 nM) unwinding of a radiolabeled FORKR DNA substrate (0.5 nM) by increasing concentrations of NCGC00015168-04. **(C)** Overlay of quantitation of gel from Fig 4A (filled circles) with graph for NCGC00185850-03 from the LOPAC^1280^ high-throughput assay (filled squares and line). The structure of NCGC00185850-03 is included in the open space of the graph. **(D)** Overlay of quantitation of gel from Fig 4B (filled circles) with graph for NCGC00015168-04 from the LOPAC^1280^ high-throughput assay (filled squares and line). The structure of NCGC00015168-04 is included in the open space of the graph.

To further validate the possibility of running the high throughput screen, a couple of inhibiting compounds from the LOPAC^1280^ library screen were tested with the GST-WRN_500-946_ helicase fragment (20 nM) in a radiometric helicase assay with the FORKR DNA substrate (0.5 nM) to verify their status as inhibitors and to determine whether the inhibition curves were similar in both the fluorescent and radiometric helicase assays (Fig 4A and 4B). Data points obtained from the radiometric assay overlaid well over the curves generated from the fluorescent data (Fig 4C and 4D).

### Fluorometric helicase activity screen of small molecule libraries to identify inhibitors of WRN-catalyzed duplex DNA unwinding

A collection of approximately 350,000 compounds is available for screening which has been assembled from multiple sources to provide diverse chemical scaffolds with known purity, identity and solubility. To perform the high-throughput screen of these compounds, we needed to produce approximately 50 milligrams of recombinant WRN helicase fragment. To accomplish this, we cloned the helicase domain fragment into several plasmids for either insect cell or bacterial expression to optimize expression and yield. We selected expression plasmids for insect cell expression with either a GST or MBP tag (image A in S1 Fig) and purified the protein (image B in S1 Fig). The GST-WRN_500-946_ fragment isolated from insect cells was used in the high throughput screen with the conditions described in Fig 2C (20 nM GST-WRN_500-946_, 100 nM FORKF DNA substrate) and a summary of the results can be found on PubChem (National Center for Biotechnology Information. PubChem BioAssay Database; Assay Identifier (AID) = 651768, https://pubchem.ncbi.nlm.nih.gov/bioassay/651768). Of the approximately 350,000 compounds tested, 0.5% were found to be active in inhibiting WRN helicase domain fragment. 1.7% of the compounds were inconclusive, and the remaining 97.8% were inactive (S2 Fig). To verify the active compounds, approximately 600 hit molecules were retested in a confirmatory screen also found on PubChem (National Center for Biotechnology Information. PubChem AID = 720497, https://pubchem.ncbi.nlm.nih.gov/bioassay/720497). Of these, a little less than four hundred compounds were confirmed as active compounds.

Two counterscreens were performed under identical conditions as the initial screen to further narrow down the active compounds for additional testing. The first was to eliminate compounds that bind to DNA (PubChem AID = 720499, https://pubchem.ncbi.nlm.nih.gov/bioassay/720499). While these compounds might inhibit WRN helicase activity they would also be predicted to nonspecifically inhibit other DNA metabolizing enzymes as well. A little less than 100 of the approximately 600 compounds tested positive for DNA binding and were not characterized further. The other counterscreen disqualified any compounds that were known to inhibit the BLM helicase domain fragment. Hits from the WRN high-throughput screen were cross-referenced with hits from the BLM screen of the same library (PubChem AID = 720503, https://pubchem.ncbi.nlm.nih.gov/bioassay/720503), and compounds that did not exhibit at least ten-fold selectivity (WRN vs. BLM) were removed as candidates.

### Validation of WRN inhibitors selected from the high-throughput screen

**Fig 5:**
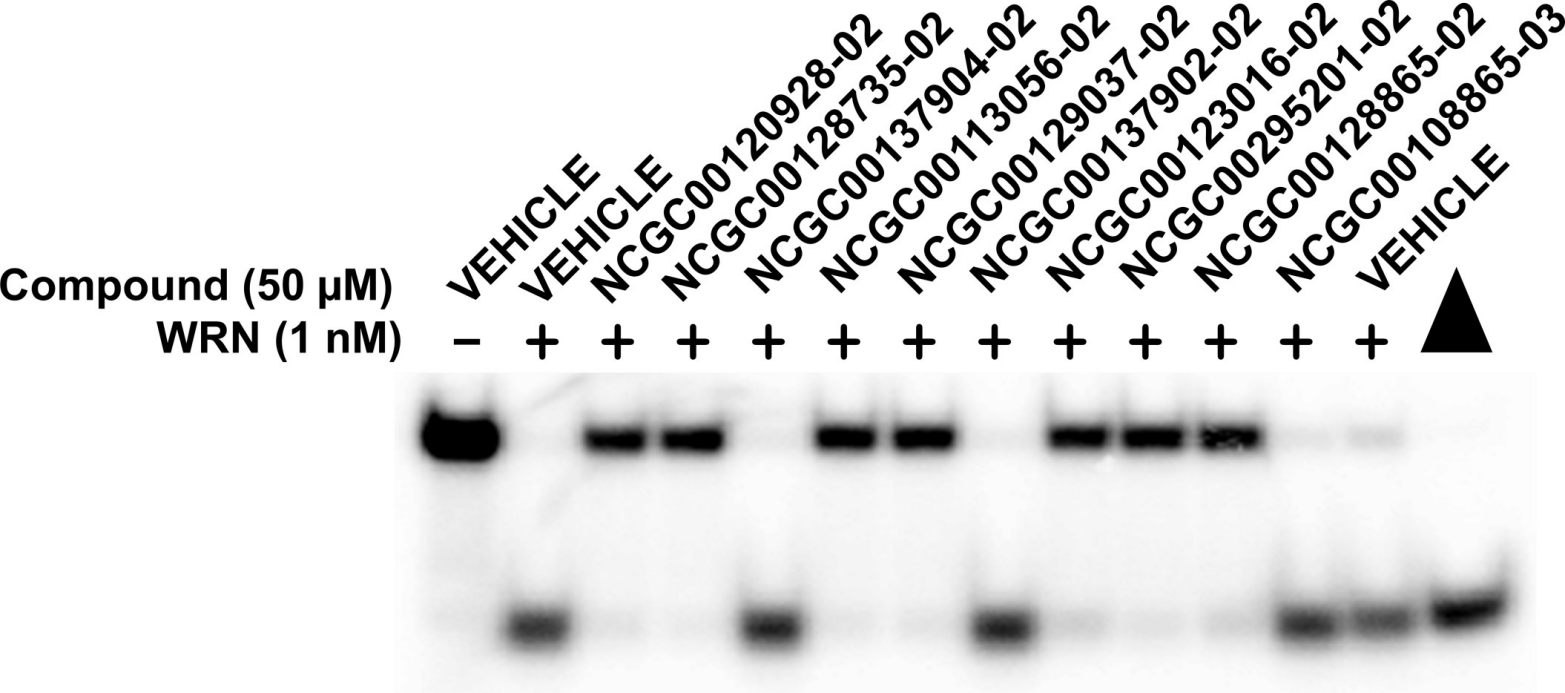
Confirmation of high-throughput screen compounds for inhibition of full-length WRN Helicase Activity. Gel image showing full-length WRN (1 nM) unwinding of the radiolabeled FORKR DNA substrate (0.5 nM) in the presence of 11 compounds at 50 μM concentration from the high-throughput screen. Vehicle is DMSO. Filled triangle is the heat-denatured DNA control.

A set of 32 compounds were selected, which represented compounds that were either inconclusive or active in one or both screens (initial and confirmatory). The compounds were tested at a single concentration of 50 μM in the radiometric helicase assay with full-length recombinant WRN protein (1 nM) and the FORKR DNA substrate (0.5 nM) as described in the Materials and Methods section. A representative gel image is included (Fig 5) showing full-length WRN unwinding of the FORKR DNA substrate in the presence of 11 of the compounds: seven of these inhibited full-length WRN protein unwinding of a forked DNA substrate, while four compounds were found to be inactive. The results for all compounds tested can be found in Table 1.

**Table 1:** Percent control WRN helicase activity with 50 μM compound.

Sample ID	% Control WRN Helicase Activity	Sample ID	% Control WRN Helicase Activity
MLS000540817-03	99	NCGC00128865-02	20
MLS000060726-02	100	NCGC00108865-03	109
NCGC00119189-02	65	NCGC00348187-01	83
NCGC00317517-02	93	NCGC00029283-02	6
MLS000582679-02	64	NCGC00063279-02	18
NCGC00307457-02	42	MLS001173584-02	84
MLS000551562-03	92	MLS002249549-02	69
MLS000715904-02	76	MLS002179845-02	93
NCGC00137930-02	99	MLS001161412-02	5
NCGC00120906-02	33	MLS002251300-02	1
NCGC00120928-02	13	NCGC00132363-04	30
NCGC00128735-02	5	MLS001139594-03	4
NCGC00137904-02	108	MLS001160745-02	8
NCGC00113056-02	10	MLS002608145-02	93
NCGC00129037-02	7	MLS002694128-02	0
NCGC00137902-02	113	MLS002608028-02	3
NCGC00123016-02	20	NCGC00183602-01	0
NCGC00295201-02	9

Shaded data represents compounds that were selected for further testing.

**Fig 6:**
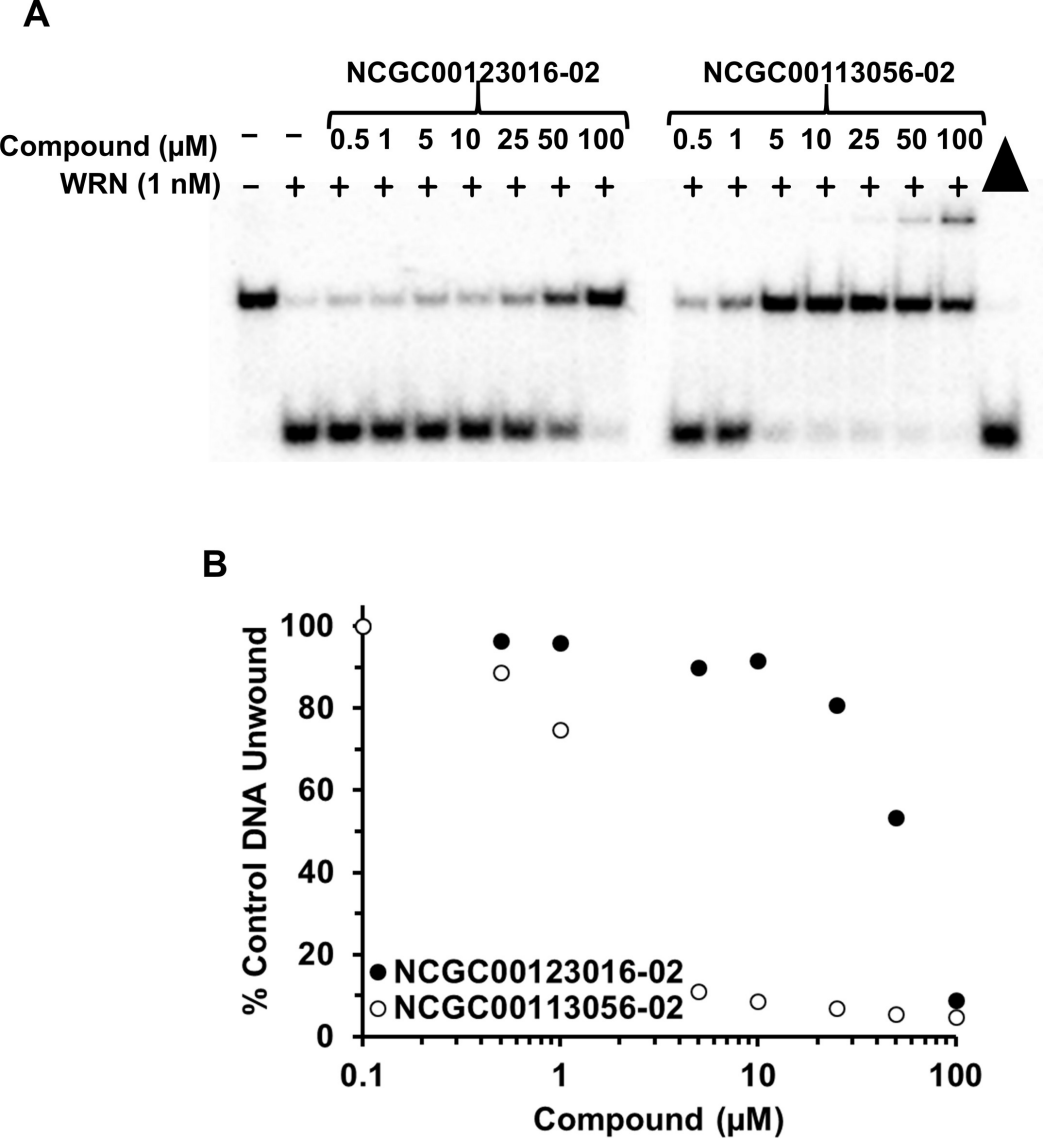
Radiometric helicase assay to determine IC_50_ values for compound inhibition of full-length WRN helicase activity. **(A)** Gel image of full-length WRN (1 nM) unwinding of the radiolabeled FORKR DNA substrate (0.5 nM) in the presence of increasing amounts of NCGC00123016-02 and NCGC00113056-02 (0–100 μM). **(B)** Quantitation of Fig 6A. Unwinding by WRN in the presence of vehicle (DMSO) is set to 100% control DNA unwinding. The x-axis is displayed in log scale.

**Fig 7:**
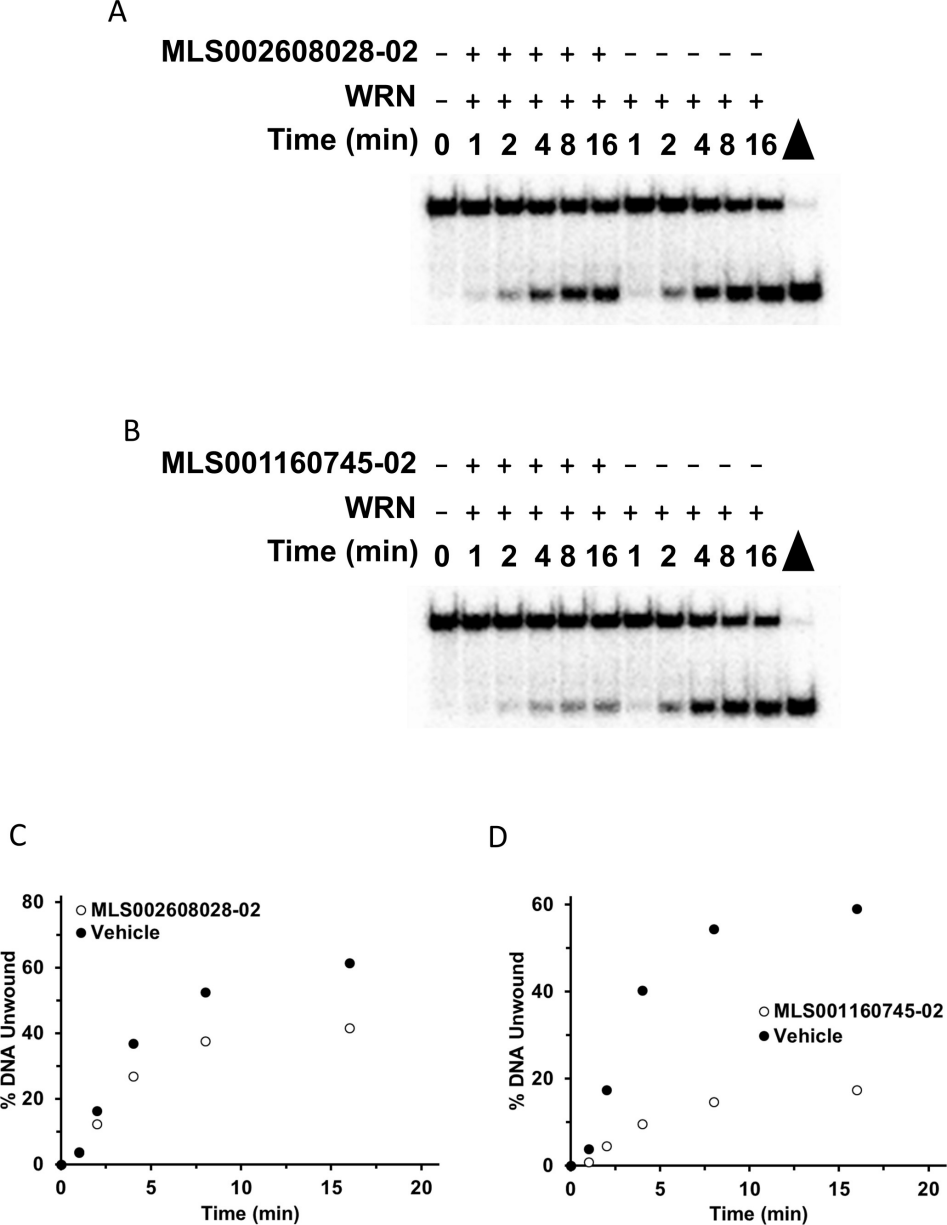
Radiometric helicase assay to determine reversibility of compound inhibition of full-length WRN helicase activity. **(A)** Gel image of full-length WRN (1 nM) unwinding kinetics (0–16 min) on the FORKR DNA substrate (0.5 nM) after dilution of MLS002608028-02 100-fold to a value 10-fold less than the IC_50_ for that compound. **(B)** Gel image of full-length WRN (1 nM) unwinding kinetics (0–16 min) on the FORKR DNA substrate (0.5 nM) after dilution of MLS001160745-02 100-fold to a value 10-fold less than IC_50_ for that compound. **(C)** Quantitation of the gel from Fig 7A. Filled circles represent WRN unwinding in the presence of vehicle (DMSO) and the open circles in the presence of MLS002608028-02. **(D)** Quantitation of the gel from Fig 7B. Filled circles represent WRN unwinding in the presence of vehicle (DMSO) and the open circles in the presence of MLS001160745-02.

The next step was to evaluate select compounds to determine IC_50_ values for inhibition and whether the compounds inhibited full-length WRN helicase activity reversibly or not. Eighteen compounds were selected based on potency of inhibition in both the screening and radiometric assays, and exclusion of compounds with undesired chemical features [22]. For each compound, we performed titrations over a wide range of concentrations with a fixed concentration of full-length WRN protein (1 nM), and quantitated unwinding as a percentage of full-length WRN unwinding of the FORKR DNA substrate (0.5 nM) in the absence of compound (Fig 6A and 6B, S3 Fig). The IC_50_ for each compound was calculated by determining the concentration of compound that resulted in a fifty percent reduction in DNA unwinding. In addition to determining an IC_50_ value for each compound, we also conducted helicase assays to determine the reversibility of inhibition by each compound tested. Details of the assay can be found in the Materials and Methods section, but briefly 100 nM WRN was incubated with each compound at a concentration 10-fold higher than its IC_50_ value. The reaction was diluted 100-fold with the addition of DNA substrate (0.5 nM final concentration) and ATP (2 mM final concentration) and unwinding was measured (Fig 7A–7D, S4 Fig). Inhibition was either classified as reversible, partially reversible, minimally reversible, or irreversible based on the ability of helicase activity to recover after dilution of the compound 100-fold. Table 2 summarizes the IC_50_ values and reversibility for each compound and includes IC_50_ values obtained from the screens for comparison.

**Table 2:** Summary of IC_50_ (μM) and reversibility results for selected compounds.

Sample ID	IC_50_ (μM)	*Reversibility	IC_50_ (μM) Assay ID 651768	IC_50_ (μM) Assay ID 720497	** IC_50_ (μM) WRN-hel-f2 protocol qHTS confirmatory
NCGC00029283-02	3	++	50.0	NT	0.45
NCGC00063279-02	12	+++	21.5	21.5	2.5
NCGC00183602-01	5	-	7.5	6.1	6.0
NCGC00295201-02	10	+++	35.4	NT	25.1
NCGC00132363-04	25	-	11.9	7.6	NA
NCGC00128735-02	5	+++	50.1	NT	28.1
NCGC00128865-02	10	++	21.1	15.2	1.8
NCGC00129037-02	9	-	16.8	13.6	12.5
NCGC00120906-02	9	-	37.6	NT	5.6
NCGC00120928-02	15	-	39.8	NT	12.5
MLS002251300-02	4	++	31.6	24.1	20
NCGC00123016-02	50	+++	26.6	NA	39.8
NCGC00113056-02	2	+	16.8	13.6	1.26
MLS002608028-02	2	+++	2.7	6.1	0.35
MLS001160745-02	11	+	44.6	9.6	22.4
MLS001139594-03	10	-	4.7	4.3	0.35

* (-) = irreversible, (+) = minimally reversible, (++) = partially reversible (+++) = reversible

** This 15 compound confirmatory assay will be deposited following publication

NA = Not Active, NT = Not Tested

**Fig 8:**
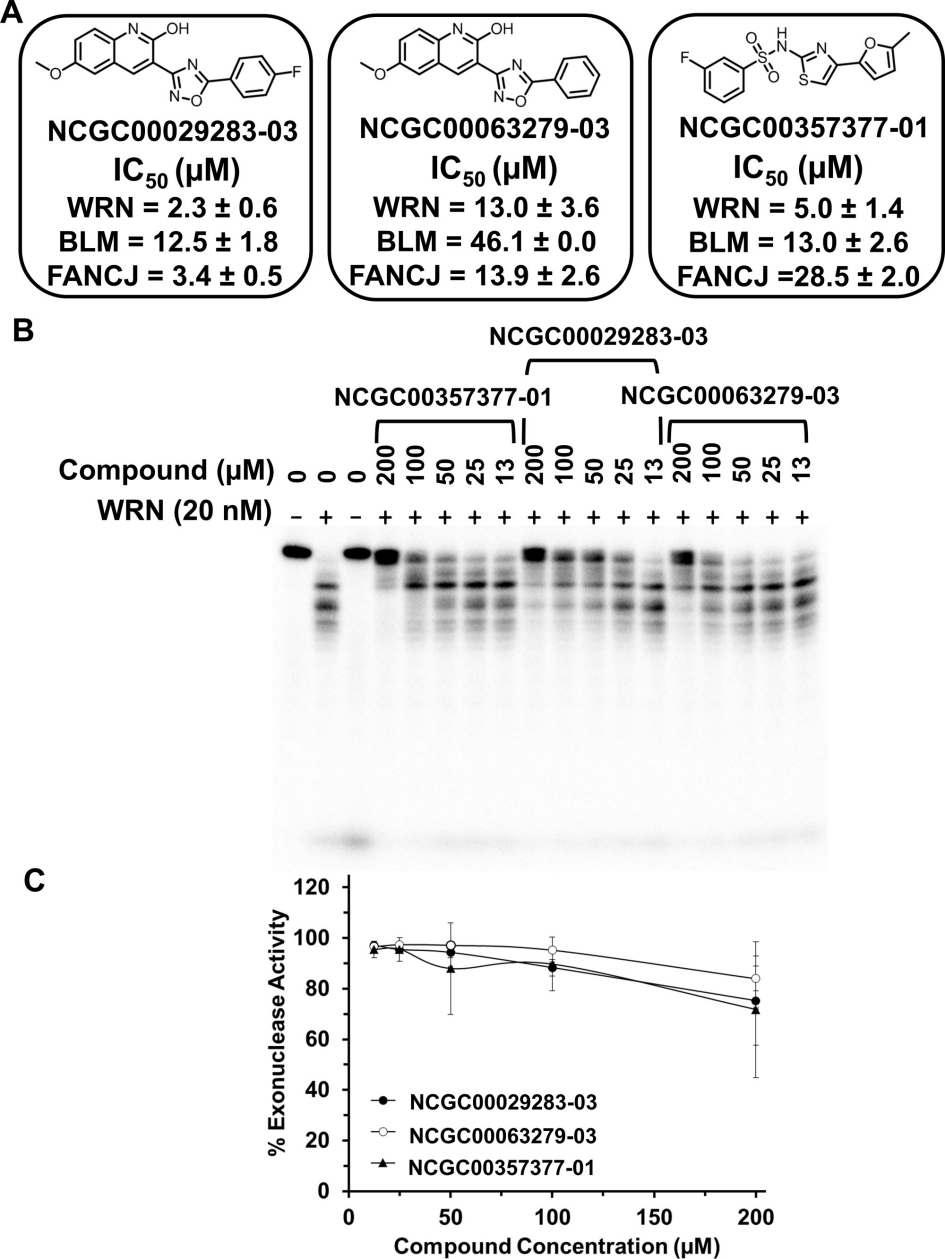
Specificity of selected compounds for inhibiting full-length WRN helicase activity. **(A)** Structures of NCGC00029283-03, NCGC00063279-03, and NCGC00357377-01 (previously referred to as MLS002251300) with IC_50_ values (μM) for each compound in the presence of WRN, BLM and FANCJ listed below. **(B)** Gel image of full-length WRN (20 nM) exonuclease activity on the radiolabeled FORKR DNA substrate (1 nM) in the presence of increasing concentrations of NCGC00029283-03, NCGC00063279-03 and NCGC00357377-01 (0–200 μM). **(C)** Quantitation of gel from Fig 8B with % exonuclease activity representing any reaction product in the ladder and inhibition of exonuclease was determined by the presence of the intact substrate as seen in the lane in the absence of protein.

Based on several factors, including IC_50_, dose-response curve, reversibility, chemical structure, activity against targets in other screens, and filters [24] such as auto-fluorescence and problematic reactive groups, the number of compounds were reduced to three (NCGC00029283, NCGC00063279, and MLS002251300, Fig 8A) which were ultimately selected for further characterization. These compounds were either resynthesized or repurified to verify their IC_50_ values as well as reversibility, upon more-thorough sourcing (MLS002251300 was resourced and purified and referred to as NCGC00357377-01, Fig 8A)

### Specificity of WRN helicase inhibitors

Three compounds identified from the cherry-picked set were tested for their effects on WRN exonuclease activity (20 nM) on the FORKR DNA substrate (1 nM) as well as DNA unwinding by full-length BLM (0.1 nM) and FANCJ (5 nM) helicases, which belong to the same Superfamily 2 as WRN [25], to assess their specificity for inhibition of WRN-catalyzed unwinding of the FORKR DNA substrate (0.5 nM). All three compounds were unable to inhibit WRN exonuclease activity in the same concentration range in which they showed activity against WRN helicase activity (Fig 8B and 8C). However, they did show some ability to inhibit exonuclease activity at a concentration of 200 μM. Of the three compounds, NCGC00357377-01 (resynthesized MLS002251300-02) displayed an IC_50_ for WRN helicase inhibition that was 6-fold less than that observed for FANCJ (Fig 8A, graph B in S5 Fig), but only 2.5-fold less than that observed for BLM (Fig 8A, graph A in S5 Fig). Previously, the BLM helicase inhibitor ML216 showed cross-reactivity for inhibition of WRN helicase activity but showed specificity for BLM inhibition in cell-based assays [16]; therefore, we pursued NCGC00357377-01 for further cell-based assays. The other two compounds (NCGC00029283-03 and NCGC00063279-03) displayed the lowest IC_50_ values for WRN helicase inhibition compared to FANCJ and BLM; however, the difference in IC_50_ values was more modest compared to NCGC00357377-01, suggesting that they may display a greater lack of specificity. Nonetheless, these two compounds were also further tested in cell-based assays to assess their effects on proliferation. In a review of PubChem data from previously-conducted screens, NCGC00357377-01 had the least number of hits in various screens of other DNA metabolizing enzymes, while NCGC00029283-03 and NCGC00063279-03 showed some activity against polymerases in high-throughput screens but no activity against BLM helicase fragment in those same screens.

### HeLa cell proliferation assays to screen WRN helicase inhibitors

**Fig 9:**
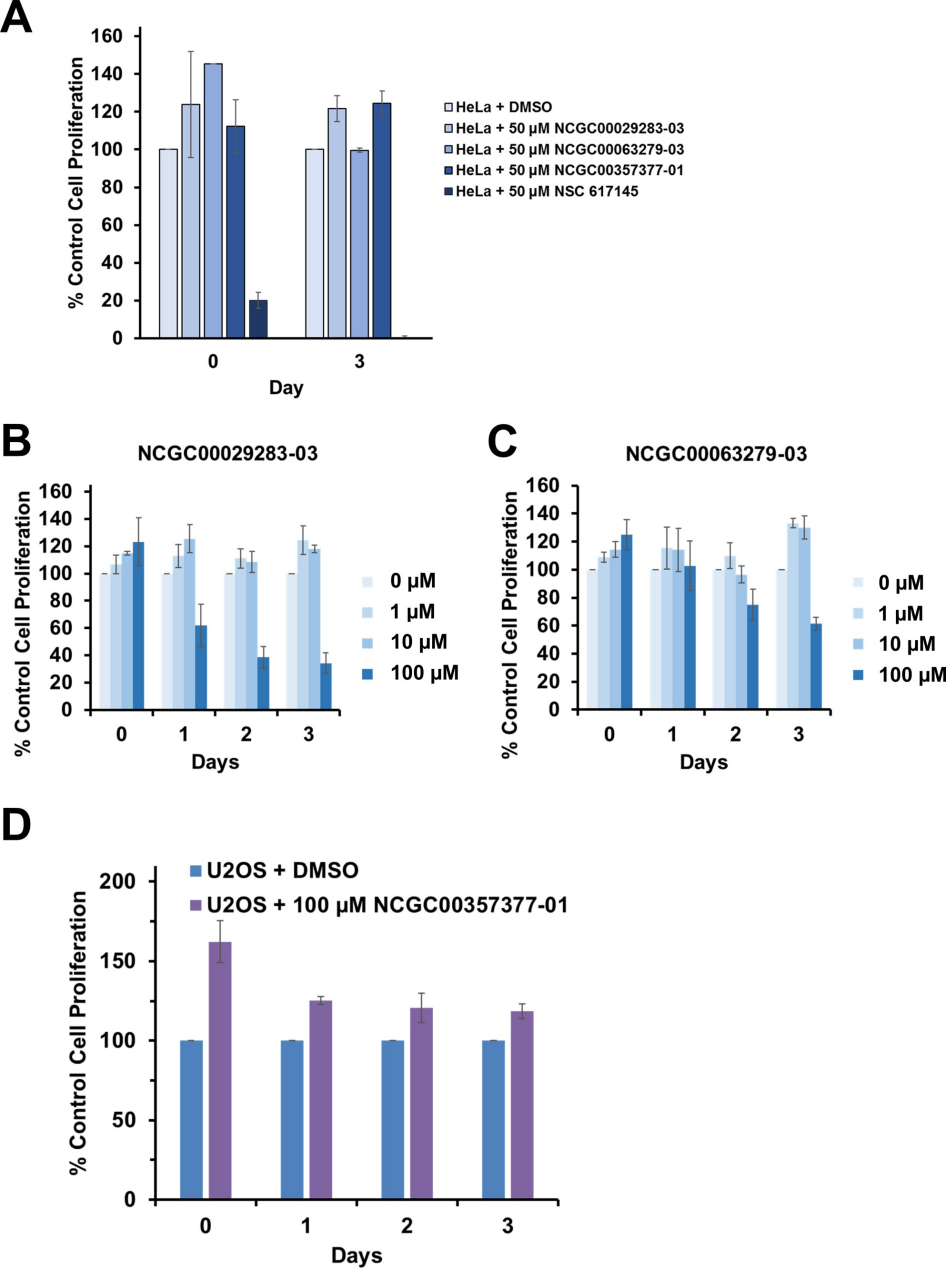
Effects of WRN helicase inhibitors on cancer cell proliferation. **(A)** Cell proliferation of HeLa 1.2.11 cells treated with 50 μM NCGC00029283-03, NCGC00063279-03, NCGC00357377-01 and NSC 617145 (positive control) or vehicle (DMSO) on Day 0 and Day 3 as measured using the WST-1 cell proliferation reagent. **(B)** Cell proliferation of U2-OS cells treated with increasing amounts of NCGC00029283-03 (0–100 μM) from Day 0 through Day 3. **(C)** Cell proliferation of U2-OS cells treated with increasing amounts of NCGC00063279-03 (0–100 μM) from Day 0 through Day 3. **(D)** Cell proliferation of U2-OS cells treated with 100 μM NCGC00357377-01 from Day 0 through Day 3.

To assess if the small molecules identified by the *in vitro* WRN helicase activity screen using fluorometric and radiometric assays were biologically active in cell-based assays, we began by examining their effects on proliferation of the human cervical cancer cell line HeLa 1.2.11. A 50 μM concentration of the selected small molecules was chosen in the initial experiment to determine if the HeLa 1.2.11 cells were sensitive at all to the potential inhibitors. HeLa 1.2.11 cells were exposed to the compound or vehicle (DMSO). To calculate % cell proliferation, the DMSO-treated cells were used as the baseline comparison. Of the three compounds tested, none showed any significant effect on HeLa 1.2.11 cell proliferation throughout the 3-day time course of viability measurements (Fig 9A). In contrast, a 50 μM concentration of the previously identified WRN helicase inhibitor NSC 617145 showed very potent inhibition of HeLa 1.2.11 cell proliferation that was detectable immediately following treatment (Fig 9A).

### WRN helicase inhibitors negatively affect proliferation of U2-OS cells

HeLa cells are characterized by reduced expression of wild-type p53 as well as an inability to increase p53 levels after DNA damage due to human papillomavirus (HPV) elements [26]. This raised the possibility that p53 status may play a role in the ability of small molecule WRN helicase inhibitors to affect cell proliferation. In addition, HeLa 1.2.11 cells are characterized by relatively long telomeres and use telomerase to maintain telomere length [27, 28]. Since WRN is implicated in the Alternative Lengthening of Telomeres (ALT) pathway [29], cells that are dependent on the ALT pathway may be more sensitive to WRN inhibitors as well. To address this possibility, we treated U2-OS osteosarcoma cells (which express wild-type p53 and utilize the ALT pathway for keeping very long telomeres) with all three compounds. In this case, we observed a 50% reduction in U2-OS cell proliferation two days after initial exposure to NCGC00029283-03 (100 μM). The effect of NCGC00029283-03 (100 μM) was also detectable after three days (Fig 9B). A 40% reduction in U2-OS cell proliferation was observed for NCGC00063279-03 (100 μM) after three days exposure (Fig 9C). NCGC00357377-01 (100 μM) did not have any effect on proliferation of U2-OS cells (Fig 9D).

## Discussion

There is considerable interest in discovering RecQ inhibitors as these compounds potentially target a key class of DNA helicases overexpressed in multiple cancers that play important roles in chemoresistance pathways used by cancer cells to overcome standard treatments [25]. High-throughput screens have been completed thus far for BLM (National Center for Biotechnology Information. PubChem BioAssay Database; Assay Identifier (AID) = 2528, https://pubchem.ncbi.nlm.nih.gov/bioassay/2528) and RECQ1 (National Center for Biotechnology Information. PubChem BioAssay Database; Assay Identifier (AID) = 2549, https://pubchem.ncbi.nlm.nih.gov/bioassay/2549). While there are published data for one BLM inhibitor and derivatives [22, 30] no other strong candidates have yet emerged from these screens. Here we have shown that a WRN helicase assay can be adapted to a high-throughput screen format using a fluorescently labeled forked DNA substrate and a GST-tagged WRN helicase domain fragment. This screen revealed some potential WRN inhibitors which were further validated and characterized to determine IC_50_ values and reversibility of inhibition. There is no obvious correlation between the two properties. For example, the average IC_50_ for the irreversible inhibitors was 12 ± 7 μM while it was 20 ± 16 μM for the completely reversible inhibitors and 11 ± 14 μM for all reversible inhibitors. Compounds with similar structures and chemistry did have similar IC_50_ values and reversibility. NCGC00029283-03 and NCGC00063279-03 have almost identical structures and have IC_50_’s of 3 μM and 12 μM, respectively, and both were at least partially reversible inhibitors. NCGC00120906-02 and NCGC00120928-02 have very similar structures as well and were both irreversible inhibitors with IC_50_ values of 9 μM and 15 μM respectively. The compounds NCGC00128735-02, NCGC00128865-02 and NCGC00129037-02 were similar structurally and while their IC_50_ values were similar as well, they were not all reversible. Irreversible inhibitors are typically less desired because they may act through nonspecific mechanisms and therefore would have a higher potential to interact with other proteins in the cell, and by doing so, may significantly complicate interpretation of WRN-specific studies that use them.

NCGC00029283, NCGC00063279, and NCGC00357377 were selected to test for specificity of inhibition, as well as bioactivity in cell lines. These compounds had chemical properties that made them good drug candidates. All three compounds had eight or less hydrogen bond acceptors and donors, a molecular mass just over 300 g/mol, and a polar surface area less than 110 A^2^. The compounds also had four or less rotatable bonds. All the compounds showed activity against either BLM or FANCJ helicase activity with an IC_50_ of 10 μM or less, so none were found to be WRN-specific inhibitors in biochemical assays. NCGC00357377 did not reduce cell proliferation of U2-OS or HeLa cell lines, while the other two compounds reduced cell proliferation in U2-OS cells only. The inability of these two compounds to reduce cell proliferation in HeLa cells may be due to several reasons: 1) the lack of functional p53 which could prevent apoptosis in these cells, 2) telomere maintenance using telomerase rather than the ALT pathway used in U2-OS cells, or 3) other unknown differences in the cell types. In contrast to the other two WRN helicase inhibitors identified in this study, NCGC00357377 was biologically inactive. A basic screen of cherry-picked compounds for cell-based biological activity early in the screening process may have filtered out compounds like NCGC003573777.

In a separate high-throughput screen, both NCGC00029283 and NCGC00063279 inhibited Hematopoietic Protein Tyrosine Phosphatase (HePTP), but neither were selected for further development as a HePTP inhibitor [31]. They are however both components of a patent describing methods for treating leukemia and myelodysplastic syndrome based on that same screen (United States Patent Application 20120095032). This is notable since leukemia cell lines from the NCI60 tumor cell line panel show higher-than-average drug sensitivity to both previously published WRN inhibitors: NSC 617145 and NSC 19630. WRN has been found to be upregulated in chronic myeloid leukemia, allowing for increased cell survival through an alternative nonhomologous end-joining pathway [32]. Recent research found that NSC 617145 and NSC 19630 can promote apoptosis in HTLV-1-transformed adult T-cell leukemia cells [33]. In addition, approximately 10% of WS patients from one study population were found to have a hematologic or lymphoid neoplasm such as acute myelogenous leukemia, T-cell leukemia or pre-leukemic marrow disorder [34]. All of this evidence points to leukemias being sensitive to compounds that regulate WRN helicase activity.

These data represent a large amount of work performed including development of scalable purified protein, assay development, assay validation, screening, counter-screening, hit validation, detailed analysis of 30 compounds, and cell-based analysis of selected compounds. Testing the library on the full-length WRN protein (with more potential binding sites), although very challenging, may yield more inhibitors that were not picked up in the helicase fragment screen. While several compounds with different IC_50_ values and reversibility were found, three were ultimately selected based on their chemistry and other considerations. Two of these compounds tested showed modest biological activity; moreover, the ability of these compounds to reversibly inhibit WRN helicase activity make them useful for assays in which transient inhibition of WRN helicase activity is required. This screening and hit triage process described here will hopefully lead to additional strategies whereby WRN catalytic function is pharmacologically modulated, and characterization of specific genetic mutant backgrounds that are most sensitive to WRN helicase inhibition, as well as chemically induced synthetic lethality with other agents that cause DNA damage or replication stress.

## Supporting information

S1 Fig Purification of helicase fragment for high-throughput screening assay. **(A)** Plasmid maps of GST- and MBP-tagged WRN helicase domain fragment expression plasmid. **(B**) Protein gel image of purified GST-tagged (HG), MBP-tagged (HM) and cleaved WRN helicase domain fragment (H, from the GST-tagged WRN helicase domain fragment). Protein standards are shown with their size in kD. (C) Protein gel image of purified recombinant BLM (full-length) with protein standards with their size in kD. (D) Protein gel image of purified recombinant WRN and FANCJ (abbreviated FJ on the figure) with protein standards with their size in kD.
(TIF)

S2 Fig Number of potential compounds derived from the high-throughput screen. qHTS assay results indicating the number of compounds that were found to be active, inactive or inconclusive from the initial screen of compounds.
(TIF)

S3 Fig IC_50_ Determination for select compounds from the high-throughput screen. Gel images of full-length WRN (1 nM) unwinding of the radiolabeled FORKR DNA substrate (0.5 nM) in the presence of increasing amounts of compounds (0–100 μM). Quantitation of gels is included for each gel. Unwinding by WRN in the presence of vehicle (DMSO) is set to 100% control DNA unwinding. The x-axis is displayed in log scale.
(PPTX)

S4 Fig Inhibition reversibility for select compounds from the high-throughput screen. Gel images of full-length WRN (1 nM) unwinding kinetics (0–16 min) of the FORKR DNA substrate (0.5 nM) after dilution of compounds 100-fold to a value 10-fold less than the IC50 for that compound and quantitation of those gels. Filled circles represent WRN unwinding in the presence of vehicle (DMSO) and the open circles in the presence of compound.
(PPTX)

S5 Fig Specificity of select WRN inhibitors for inhibition of other human helicases. **(A)** Quantitation of full-length BLM helicase (0.1 nM) activity on the FORKR DNA substrate (0.5 nM) with increasing concentration of each compound (0–100 μM). **(B)** Quantitation of full-length FANCJ (5 nM) helicase activity on the FORKR DNA substrate (0.5 nM) with increasing concentration of each compound (0–100 μM).
(TIF)

S1 Table Structure and supplier information for study compounds. Detailed information about compounds tested in this study including SMILE structures, NCGC ID numbers, batch numbers, suppliers and suppliers’ ID numbers.
(XLSX)
