# Supplementary figures and images for: A high-throughput screen to identify novel small molecule inhibitors of the Werner Syndrome Helicase-Nuclease (WRN)
Source: PLoS One. 2019 Jan 9;14(1):e0210525. doi: 10.1371/journal.pone.0210525 (PMC6326523; doi:10.1371/journal.pone.0210525)

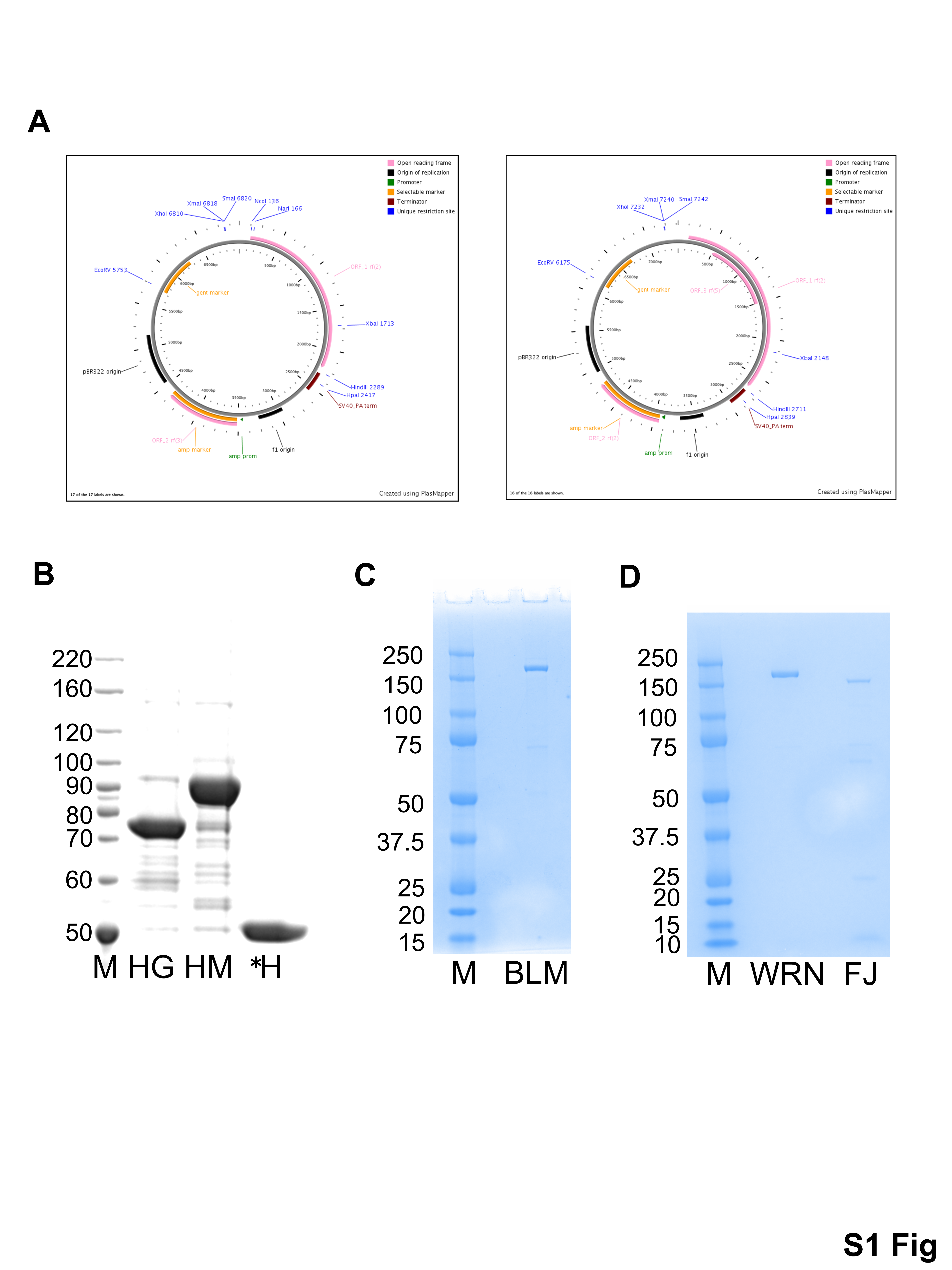

Supplement: S1 Fig — (A) Plasmid maps of GST- and MBP-tagged WRN helicase domain fragment expression plasmid. (B) Protein gel image of purified GST-tagged (HG), MBP-tagged (HM) and cleaved WRN helicase domain fragment (H, from the GST-tagged WRN helicase domain fragment). Protein standards are shown with their size in kD. (C) Protein gel image of purified recombinant BLM (full-length) with protein standards with their size in kD. (D) Protein gel image of purified recombinant WRN and FANCJ (abbreviated FJ on the figure) with protein standards with their size in kD. (TIF) [file pone.0210525.s001.TIF]

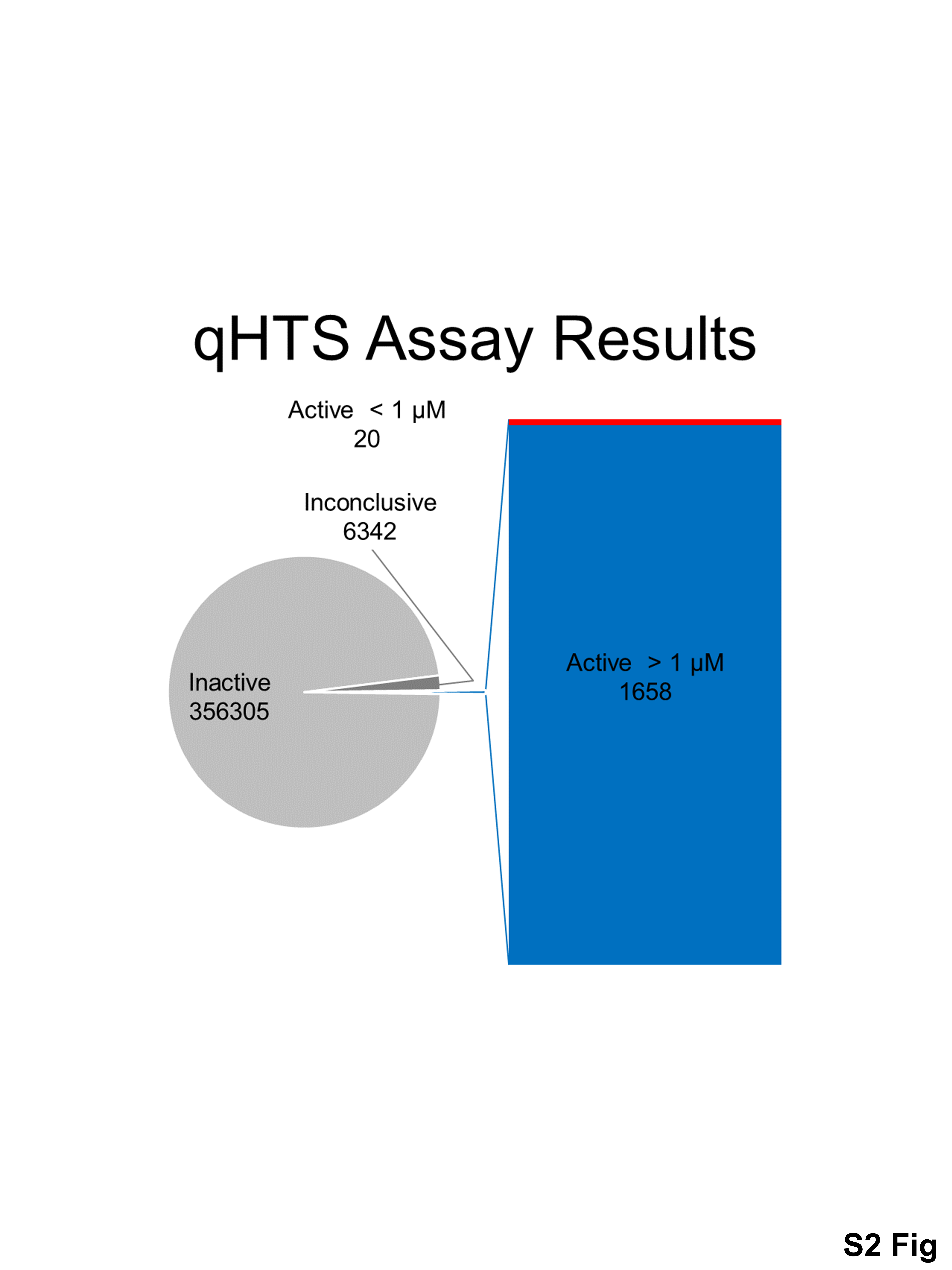

Supplement: S2 Fig — qHTS assay results indicating the number of compounds that were found to be active, inactive or inconclusive from the initial screen of compounds. (TIF) [file pone.0210525.s002.TIF]

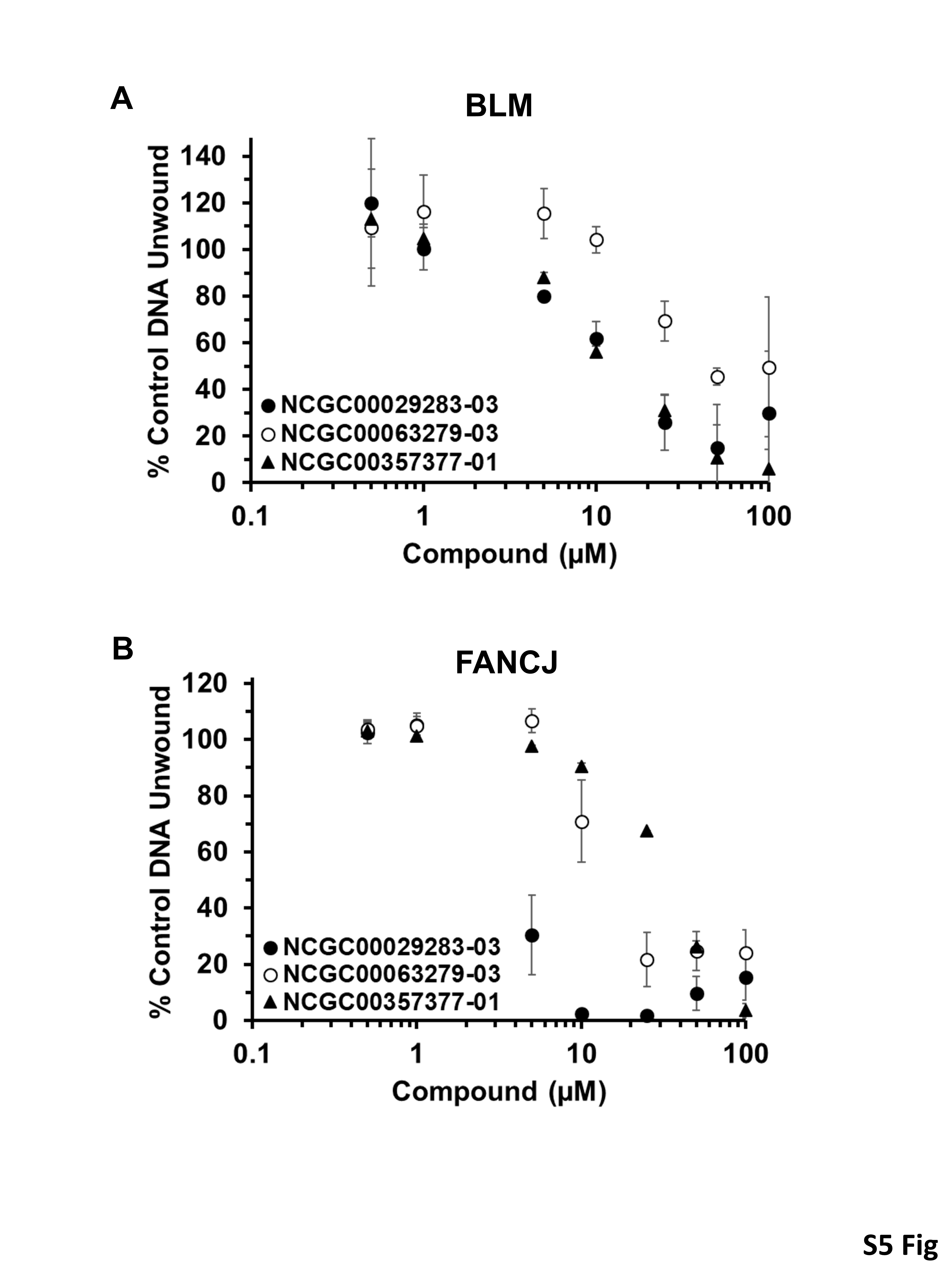

Supplement: S5 Fig — (A) Quantitation of full-length BLM helicase (0.1 nM) activity on the FORKR DNA substrate (0.5 nM) with increasing concentration of each compound (0–100 μM). (B) Quantitation of full-length FANCJ (5 nM) helicase activity on the FORKR DNA substrate (0.5 nM) with increasing concentration of each compound (0–100 μM). (TIF) [file pone.0210525.s005.TIF]
