# Supplementary material for: A high-throughput screen to identify novel small molecule inhibitors of the Werner Syndrome Helicase-Nuclease (WRN)
Source: PLoS One. 2019 Jan 9;14(1):e0210525. doi: 10.1371/journal.pone.0210525 (PMC6326523; doi:10.1371/journal.pone.0210525)
Supplement: S3 Fig — Gel images of full-length WRN (1 nM) unwinding of the radiolabeled FORKR DNA substrate (0.5 nM) in the presence of increasing amounts of compounds (0–100 μM). Quantitation of gels is included for each gel. Unwinding by WRN in the presence of vehicle (DMSO) is set to 100% control DNA unwinding. The x-axis is displayed in log scale. (PPTX) [file pone.0210525.s003.pptx]

## Slide 1
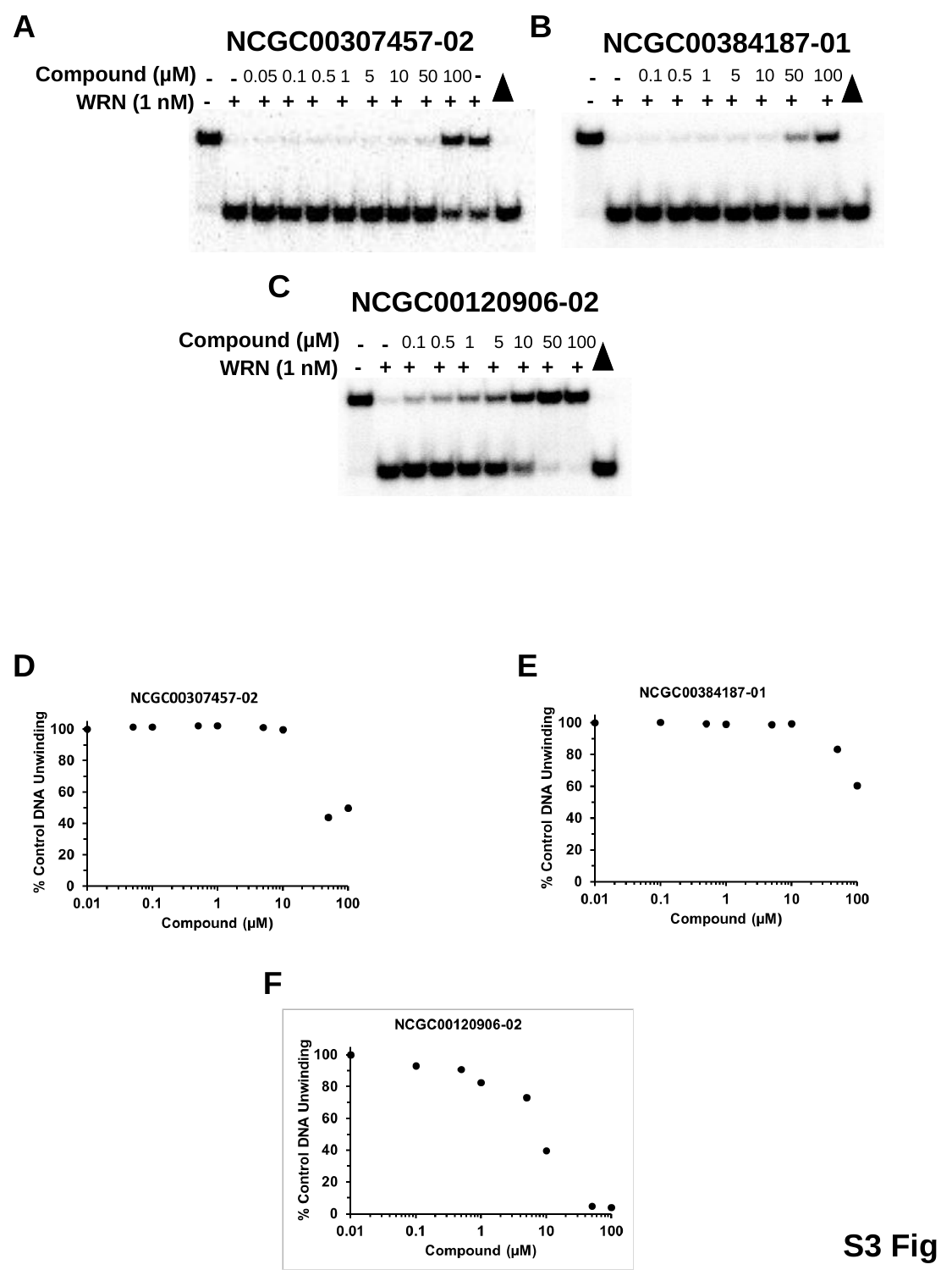

A
B
NCGC00307457-02
NCGC00384187-01
 Compound (µM)
 WRN (1 nM)
 0.1 0.5 1 5 10 50 100
 0.05 0.1 0.5 1 5 10 50 100
-
- -
- -
- + + + + + + + +
- + + + + + + + + + +
C
NCGC00120906-02
 Compound (µM)
 WRN (1 nM)
 0.1 0.5 1 5 10 50 100
- -
- + + + + + + + +
E
D
F
S3 Fig

## Slide 2
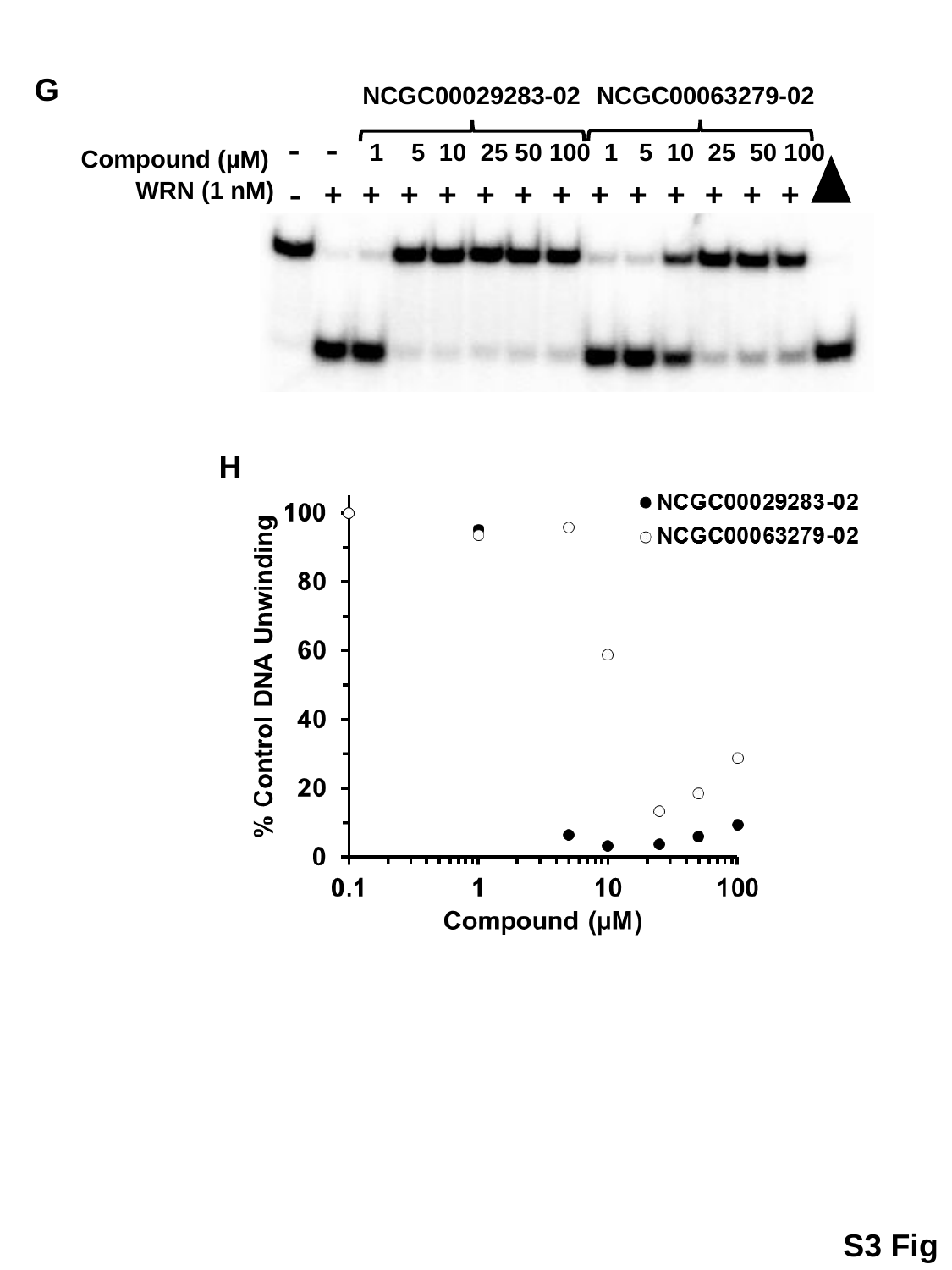

G
NCGC00063279-02
NCGC00029283-02
- -
 Compound (µM)
 WRN (1 nM)
 1 5 10 25 50 100 1 5 10 25 50 100
- + + + + + + + + + + + + +
H
S3 Fig

## Slide 3
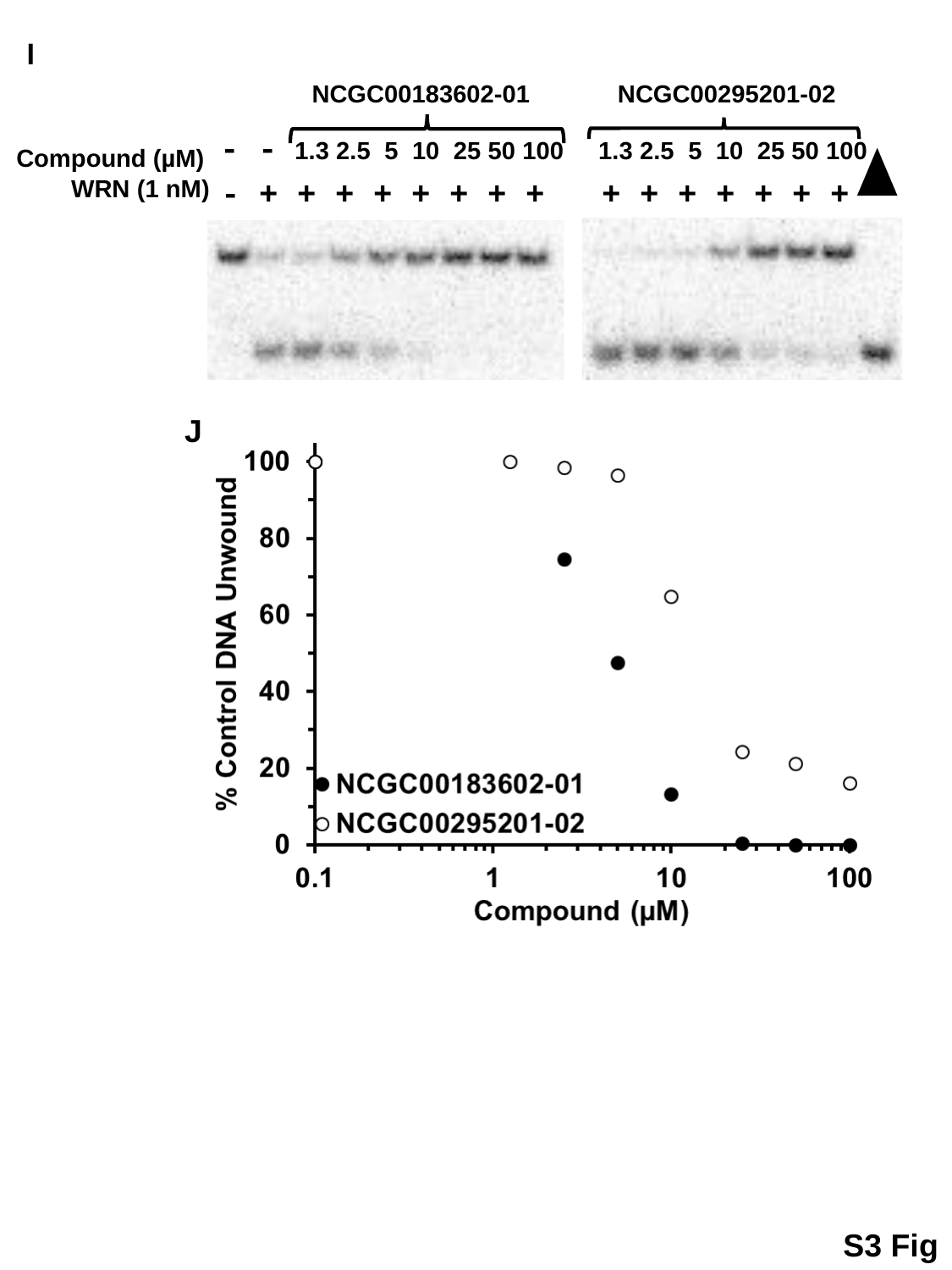

I
NCGC00295201-02
NCGC00183602-01
- -
 Compound (µM)
 WRN (1 nM)
 1.3 2.5 5 10 25 50 100 1.3 2.5 5 10 25 50 100
- + + + + + + + + + + + + + + +
J
S3 Fig

## Slide 4
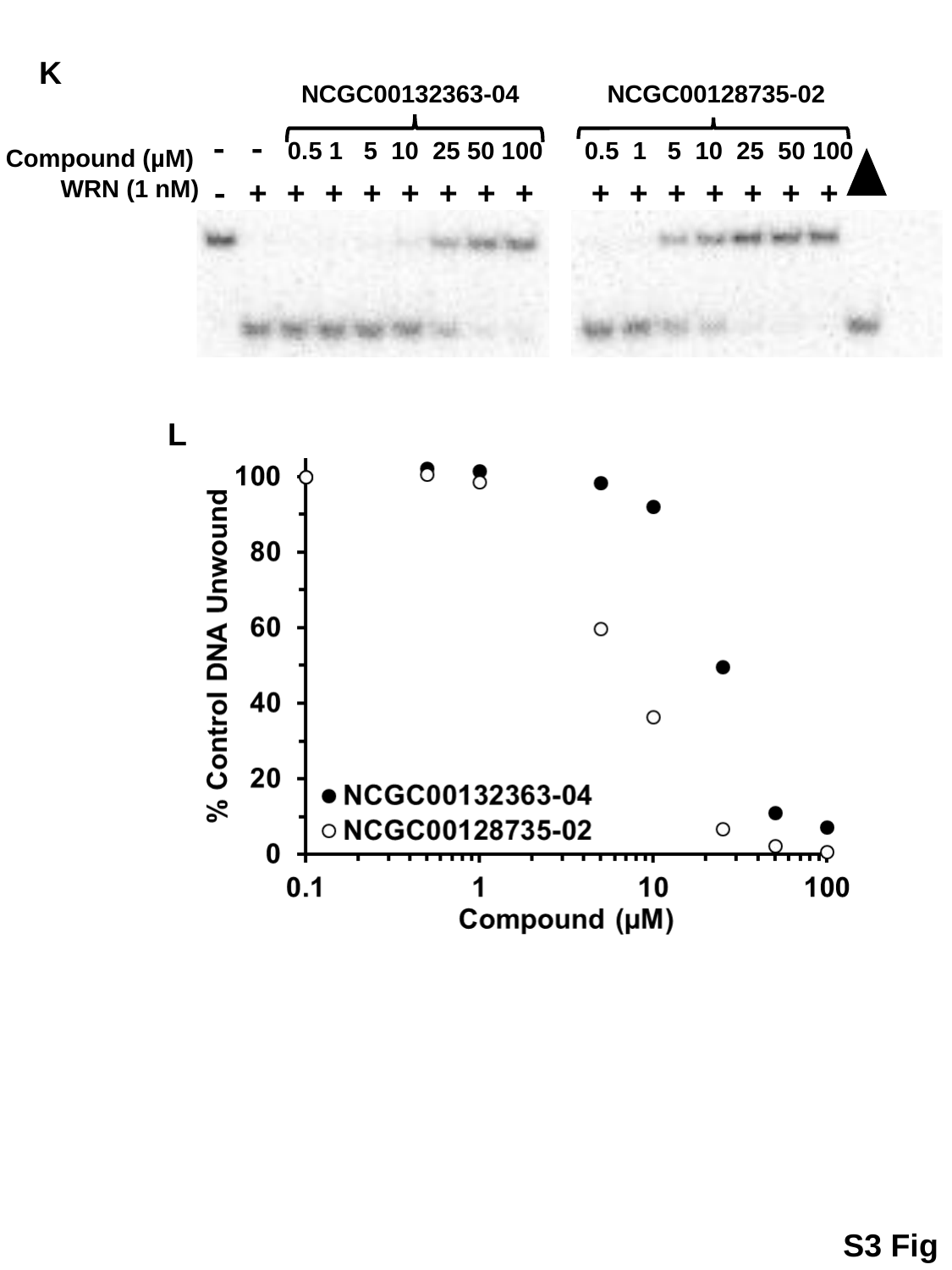

K
NCGC00128735-02
NCGC00132363-04
- -
 Compound (µM)
 WRN (1 nM)
 0.5 1 5 10 25 50 100 0.5 1 5 10 25 50 100
- + + + + + + + + + + + + + + +
L
S3 Fig

## Slide 5
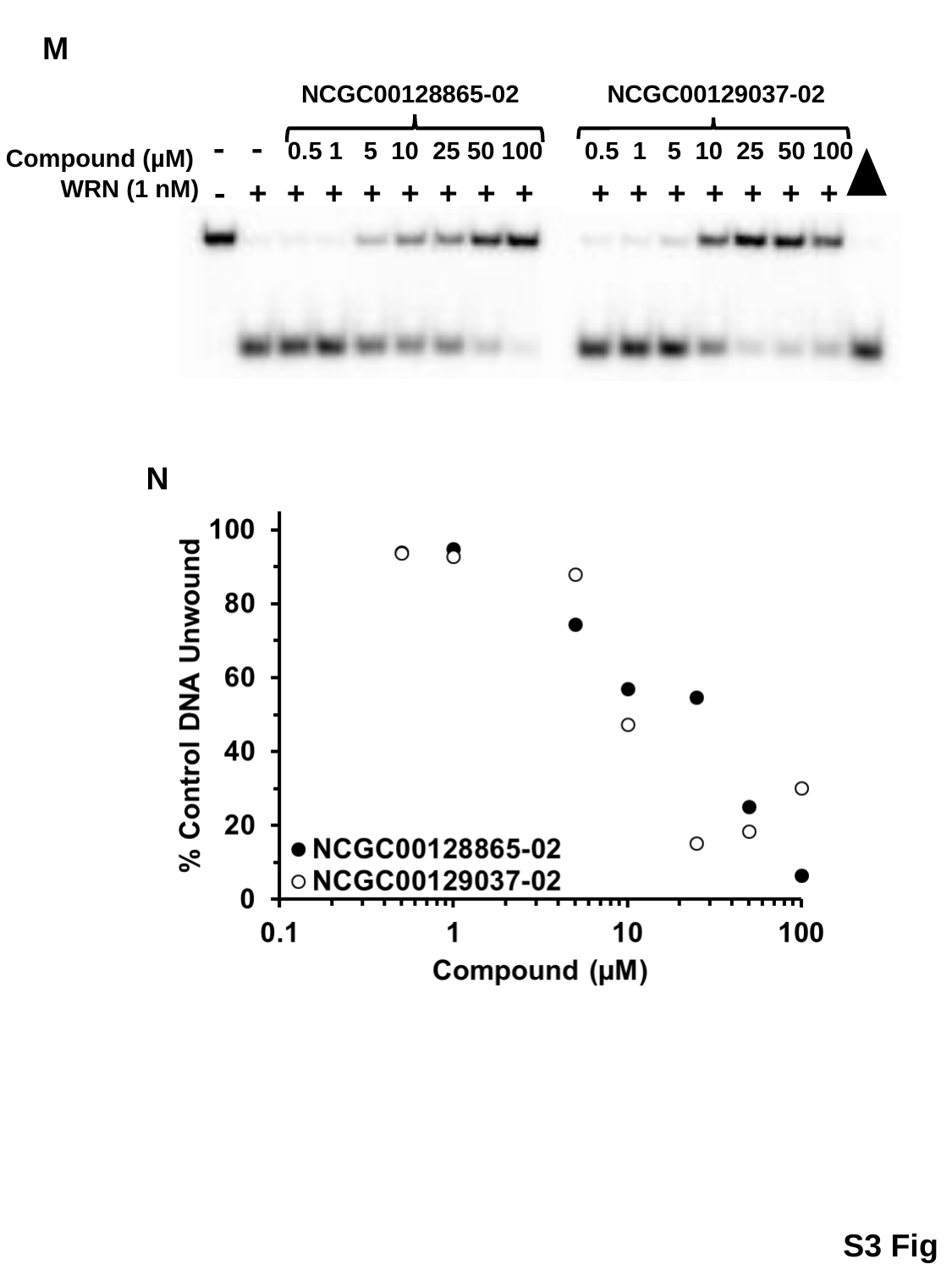

M
NCGC00129037-02
NCGC00128865-02
- -
 Compound (µM)
 WRN (1 nM)
 0.5 1 5 10 25 50 100 0.5 1 5 10 25 50 100
- + + + + + + + + + + + + + + +
N
S3 Fig

## Slide 6
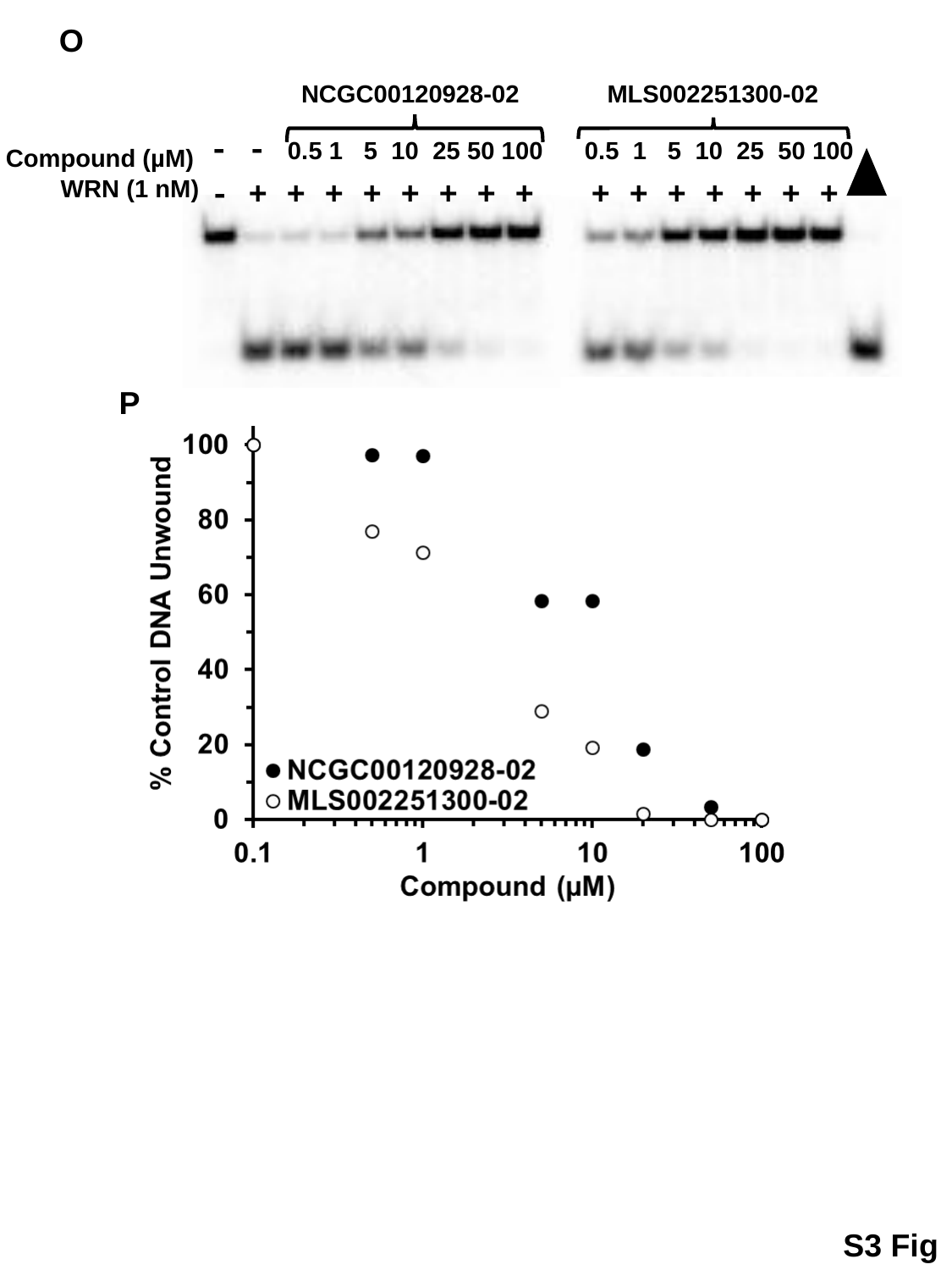

O
MLS002251300-02
NCGC00120928-02
- -
 Compound (µM)
 WRN (1 nM)
 0.5 1 5 10 25 50 100 0.5 1 5 10 25 50 100
- + + + + + + + + + + + + + + +
P
S3 Fig

## Slide 7
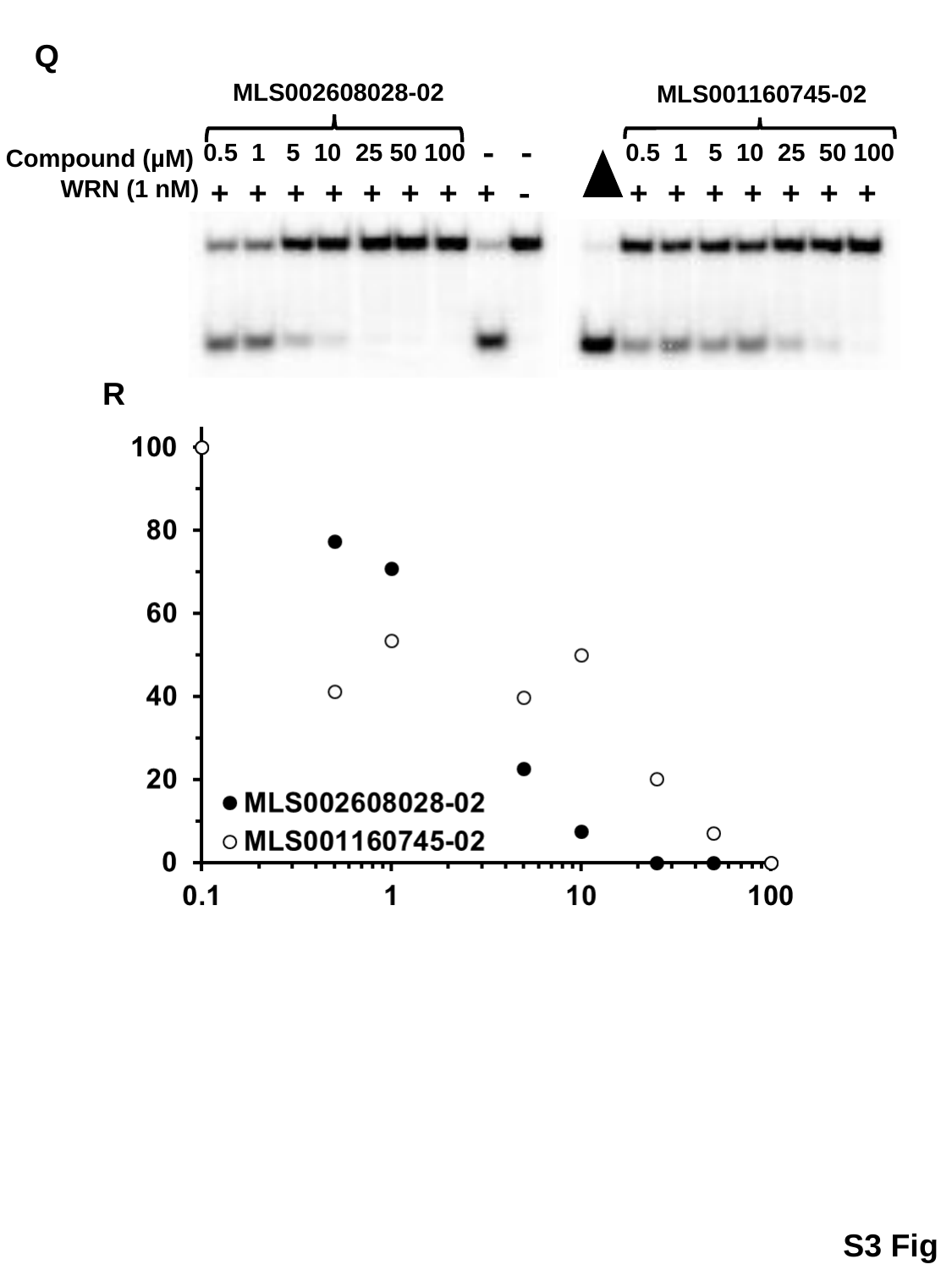

Q
MLS002608028-02
MLS001160745-02
- -
 Compound (µM)
 WRN (1 nM)
 0.5 1 5 10 25 50 100 0.5 1 5 10 25 50 100
+ + + + + + + + - + + + + + + +
R
S3 Fig

## Slide 8
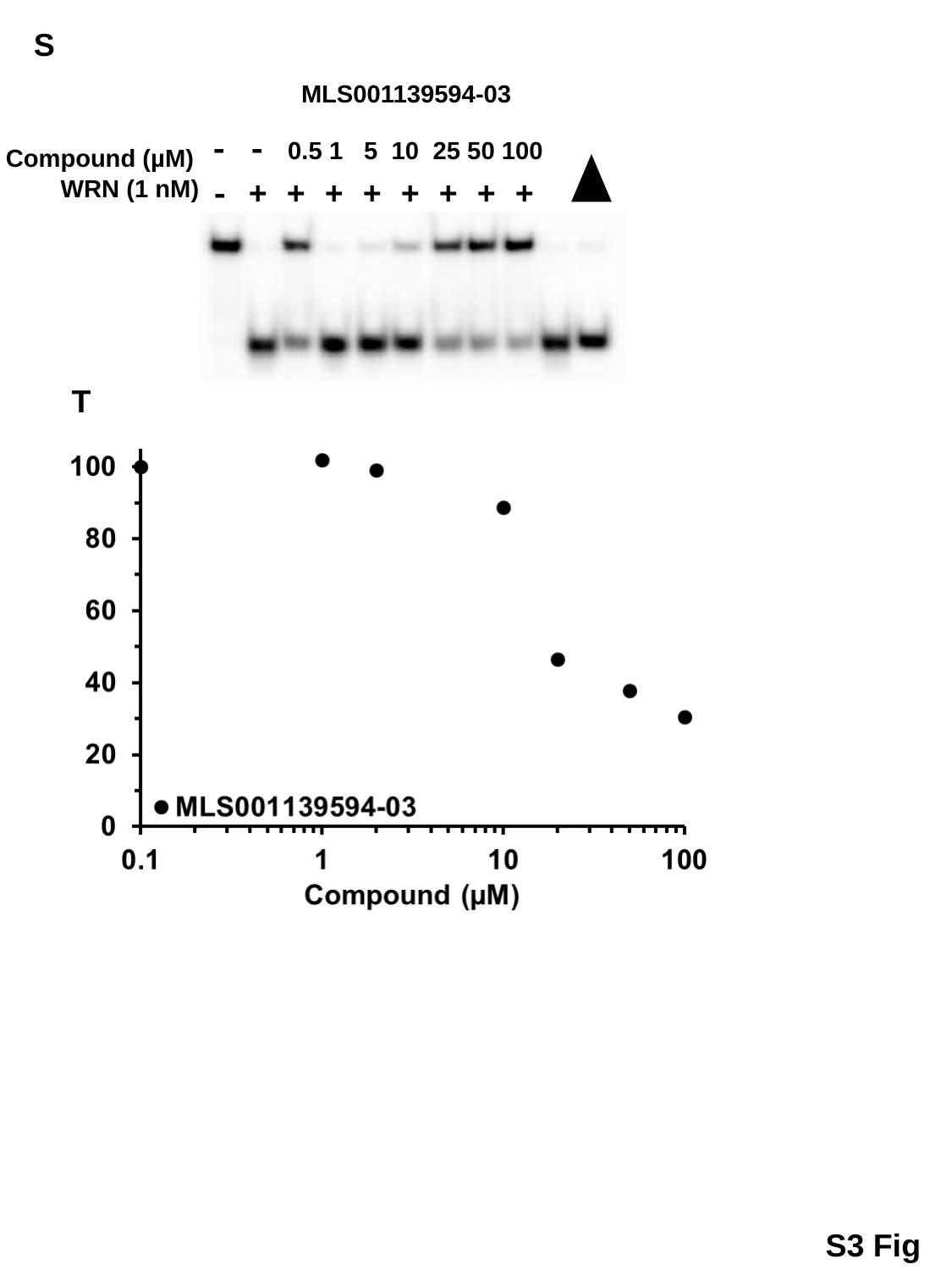

S
MLS001139594-03
- -
 Compound (µM)
 WRN (1 nM)
 0.5 1 5 10 25 50 100
- + + + + + + + +
T
S3 Fig
