# Supplementary material for: A high-throughput screen to identify novel small molecule inhibitors of the Werner Syndrome Helicase-Nuclease (WRN)
Source: PLoS One. 2019 Jan 9;14(1):e0210525. doi: 10.1371/journal.pone.0210525 (PMC6326523; doi:10.1371/journal.pone.0210525)
Supplement: S4 Fig — Gel images of full-length WRN (1 nM) unwinding kinetics (0–16 min) of the FORKR DNA substrate (0.5 nM) after dilution of compounds 100-fold to a value 10-fold less than the IC50 for that compound and quantitation of those gels. Filled circles represent WRN unwinding in the presence of vehicle (DMSO) and the open circles in the presence of compound. (PPTX) [file pone.0210525.s004.pptx]

## Slide 1
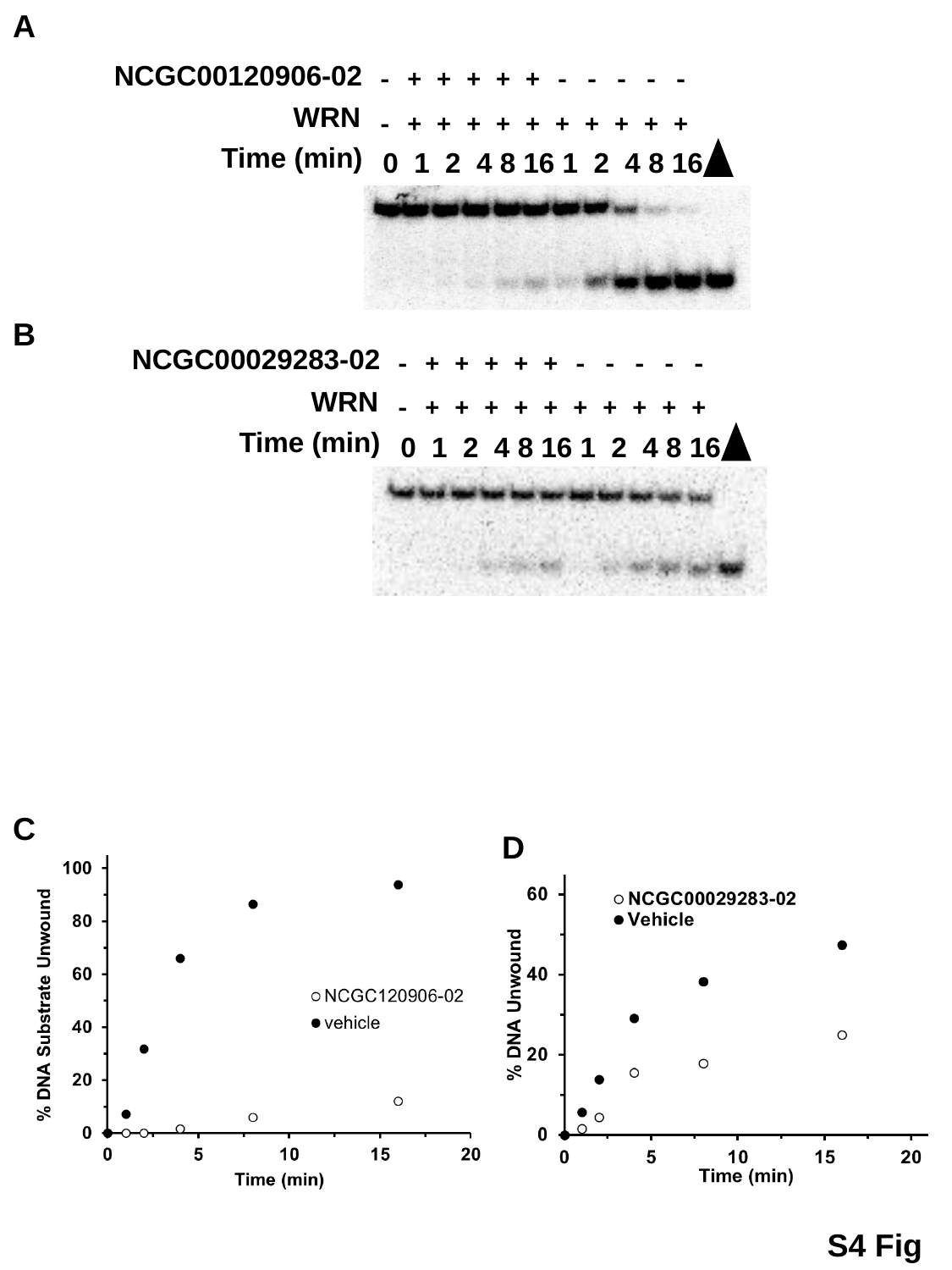

A
NCGC00120906-02
- + + + + + - - - - -
WRN
- + + + + + + + + + +
Time (min)
0 1 2 4 8 16 1 2 4 8 16
B
NCGC00029283-02
- + + + + + - - - - -
WRN
- + + + + + + + + + +
Time (min)
0 1 2 4 8 16 1 2 4 8 16
C
D
S4 Fig

## Slide 2
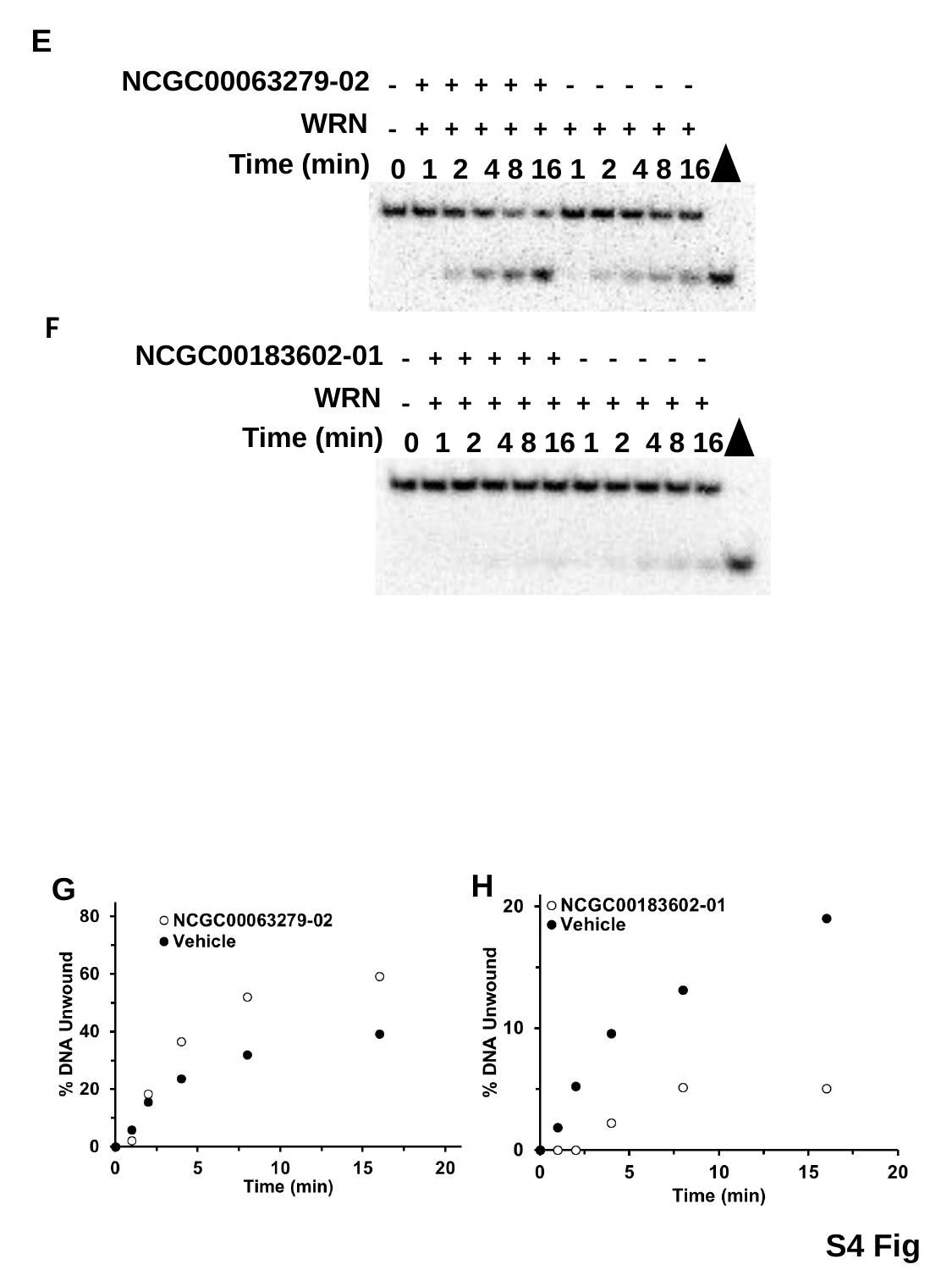

E
NCGC00063279-02
- + + + + + - - - - -
WRN
- + + + + + + + + + +
Time (min)
0 1 2 4 8 16 1 2 4 8 16
F
NCGC00183602-01
- + + + + + - - - - -
WRN
- + + + + + + + + + +
Time (min)
0 1 2 4 8 16 1 2 4 8 16
H
G
S4 Fig

## Slide 3
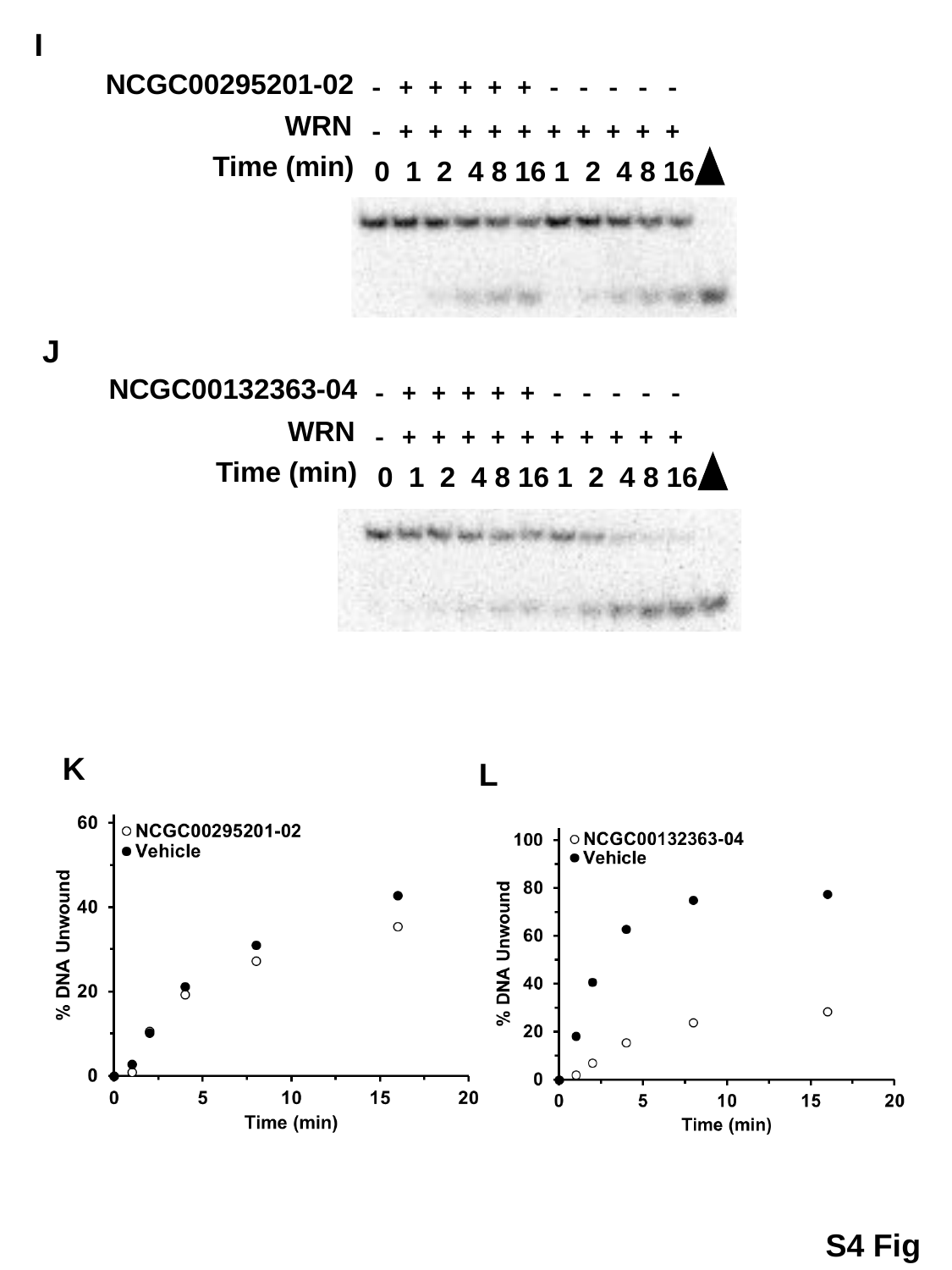

I
NCGC00295201-02
- + + + + + - - - - -
WRN
- + + + + + + + + + +
Time (min)
0 1 2 4 8 16 1 2 4 8 16
J
NCGC00132363-04
- + + + + + - - - - -
WRN
- + + + + + + + + + +
Time (min)
0 1 2 4 8 16 1 2 4 8 16
K
L
S4 Fig

## Slide 4
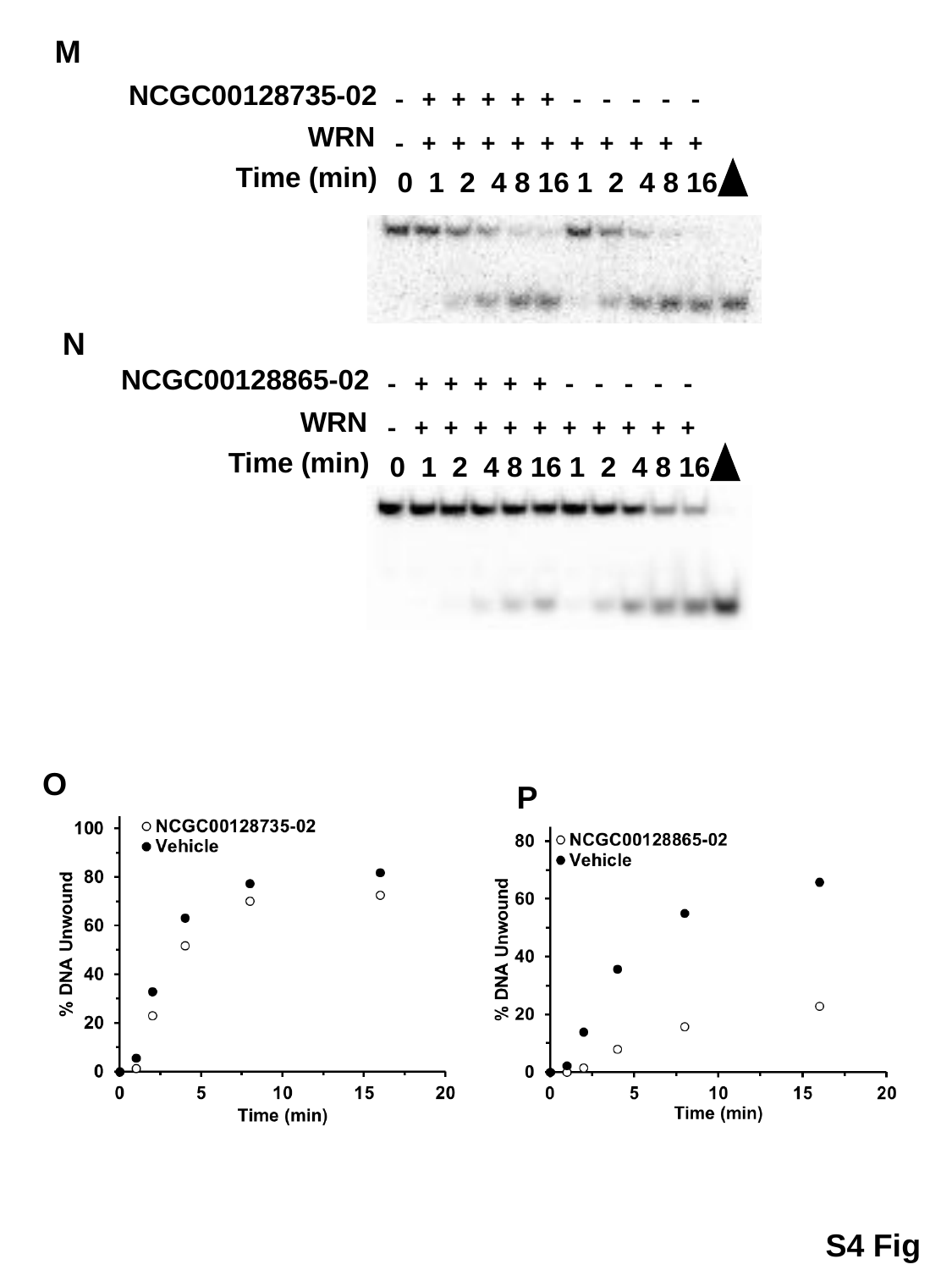

M
NCGC00128735-02
- + + + + + - - - - -
WRN
- + + + + + + + + + +
Time (min)
0 1 2 4 8 16 1 2 4 8 16
N
NCGC00128865-02
- + + + + + - - - - -
WRN
- + + + + + + + + + +
Time (min)
0 1 2 4 8 16 1 2 4 8 16
O
P
S4 Fig

## Slide 5
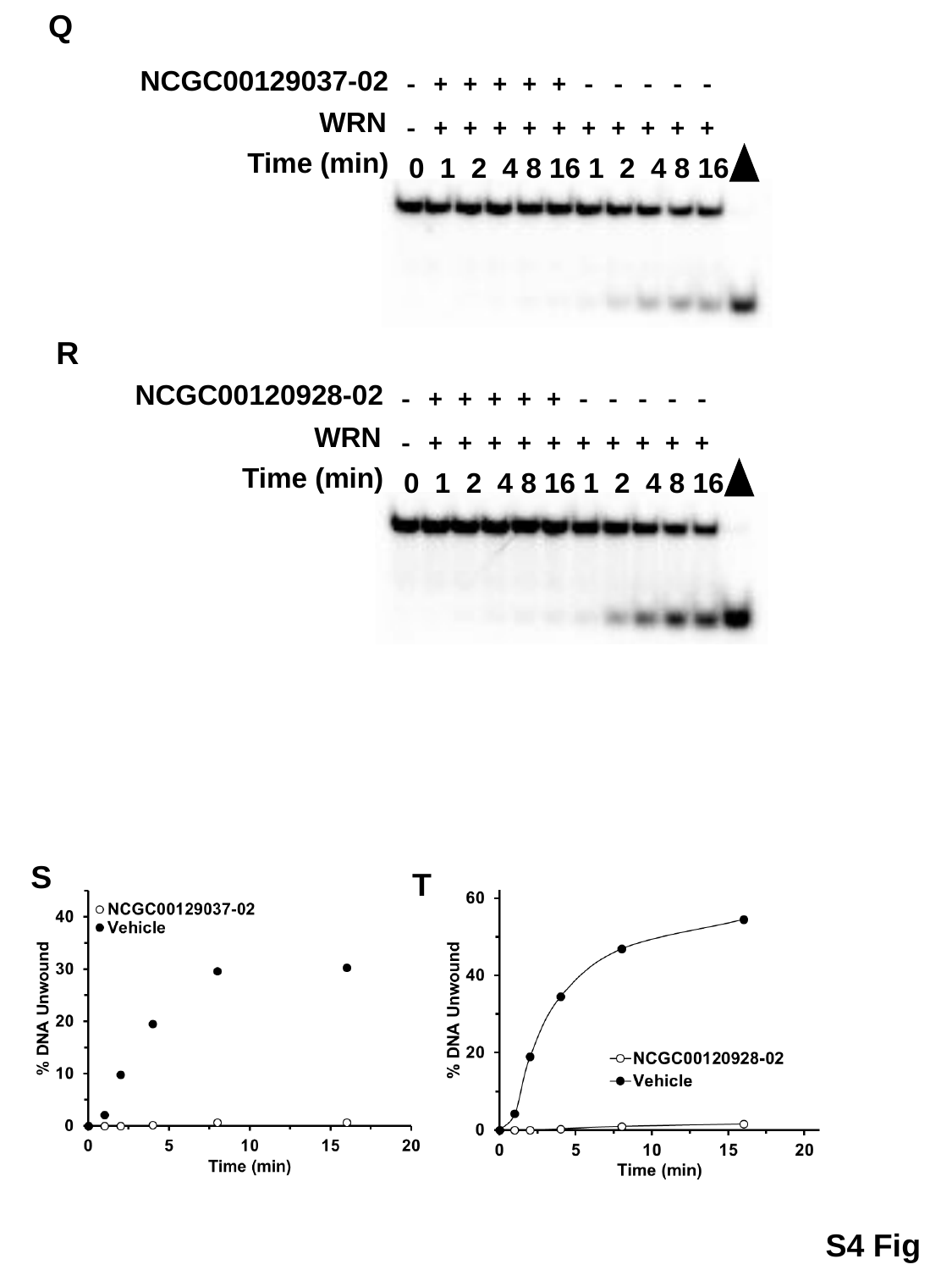

Q
NCGC00129037-02
- + + + + + - - - - -
WRN
- + + + + + + + + + +
Time (min)
0 1 2 4 8 16 1 2 4 8 16
R
NCGC00120928-02
- + + + + + - - - - -
WRN
- + + + + + + + + + +
Time (min)
0 1 2 4 8 16 1 2 4 8 16
S
T
S4 Fig

## Slide 6
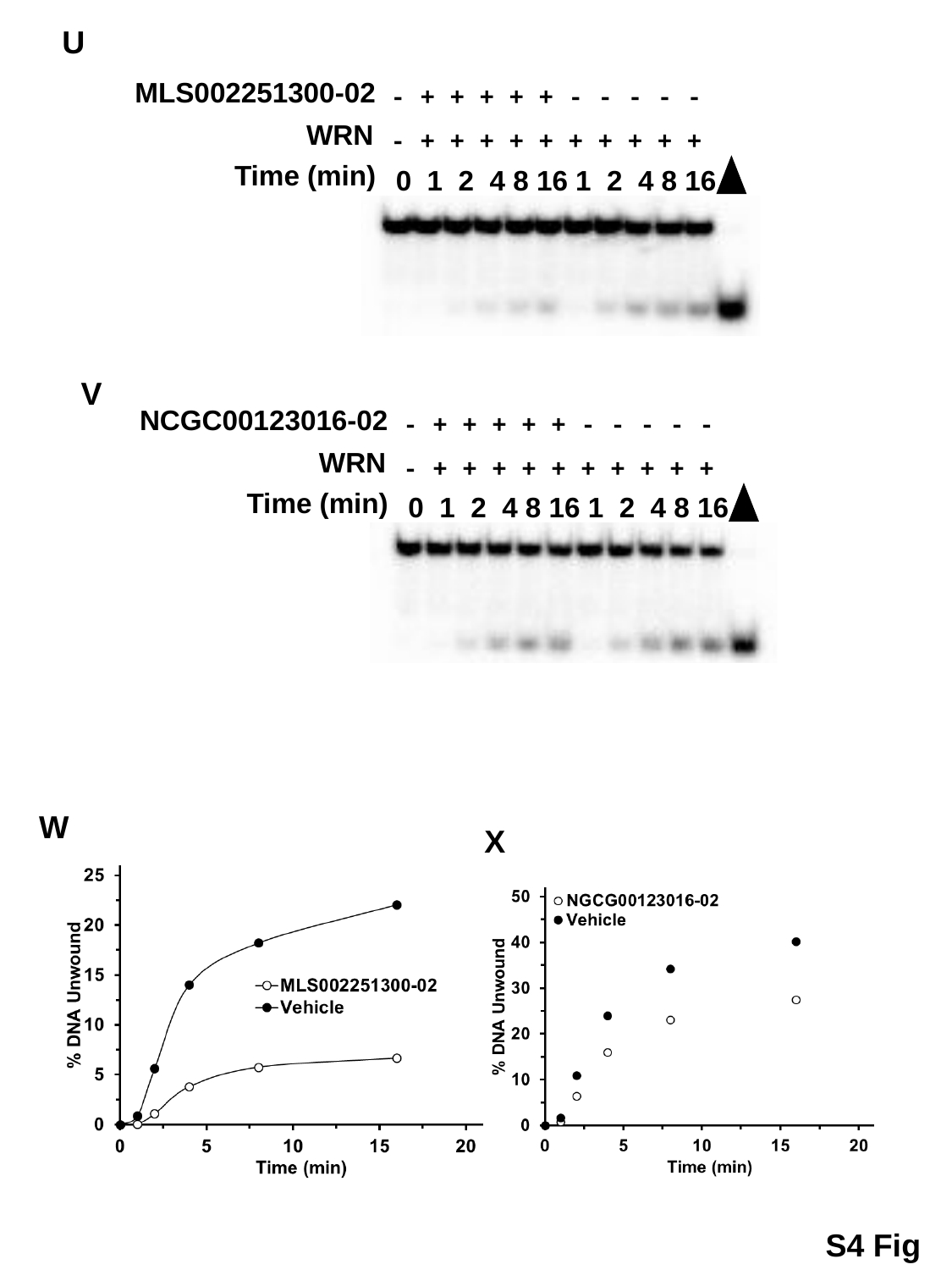

U
MLS002251300-02
- + + + + + - - - - -
WRN
- + + + + + + + + + +
Time (min)
0 1 2 4 8 16 1 2 4 8 16
V
NCGC00123016-02
- + + + + + - - - - -
WRN
- + + + + + + + + + +
Time (min)
0 1 2 4 8 16 1 2 4 8 16
W
X
S4 Fig

## Slide 7
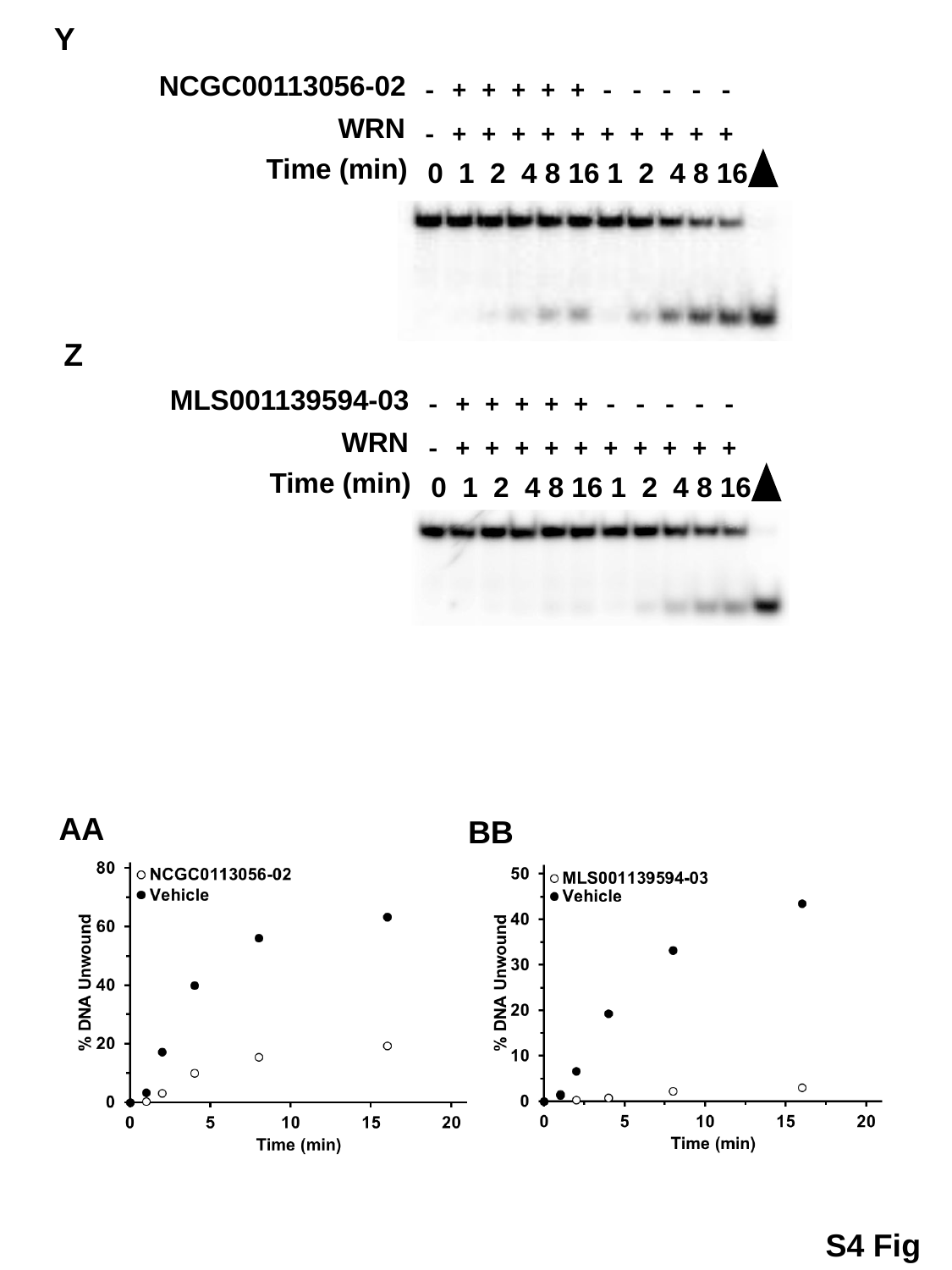

Y
NCGC00113056-02
- + + + + + - - - - -
WRN
- + + + + + + + + + +
Time (min)
0 1 2 4 8 16 1 2 4 8 16
Z
MLS001139594-03
- + + + + + - - - - -
WRN
- + + + + + + + + + +
Time (min)
0 1 2 4 8 16 1 2 4 8 16
AA
BB
S4 Fig
